# A lateral hypothalamic neuronal population expressing leptin receptors counteracts anxiety to enable adaptive behavioral responses

**DOI:** 10.1038/s41593-025-02078-y

**Published:** 2025-10-20

**Authors:** Rebecca Figge-Schlensok, Anne Petzold, Nele Hugger, Alisa Bakhareva, Ali Taleb Abdallah, Chantal Wissing, Hanna Elin van den Munkhof, Marla Yasmin Witt, Deema Imad Awad, Tatiana Korotkova

**Affiliations:** 1 Institute for Systems Physiology, Faculty of Medicine, University of Cologne/University Clinic Cologne, Cologne, Germany; 2 Cluster of Excellence Cellular Stress Responses in Aging-Associated Diseases (CECAD), University of Cologne, Cologne, Germany; 3 European Neuroscience Institute, A Joint Initiative of the University Medical Center Göttingen and the Max Planck Institute for Multidisciplinary Sciences, Göttingen, Germany; 4 Institute of Medical Statistics and Computational Biology, Faculty of Medicine, University of Cologne, Cologne, Germany; 5 Center of Molecular Medicine Cologne (CMMC), University of Cologne, Cologne, Germany

**Keywords:** Neural circuits, Hypothalamus, Prefrontal cortex, Anxiety

## Abstract

Neuronal mechanisms that facilitate adaptive strategies to enable an animal to overcome anxiety in threatening situations remain unknown. Using single-cell calcium imaging and cell-type-specific activity manipulations in behaving mice, we identified leptin-sensitive neuronal subpopulations in the lateral hypothalamus (LH; LepR^LH^) that encode anxiogenic stimuli. In high-anxiety animals, LepR^LH^ neurons differentiated poorly among anxiogenic stimuli and were inhibited by input from the prefrontal cortex. The activity of LepR^LH^ neurons predicted the anxiety level of individual animals, and the activation of LepR^LH^ neurons enabled adaptive responses under anxiogenic conditions—exploration of new terrain, eating despite anxiogenic environment and limiting maladaptive excessive locomotion in an anorexia nervosa disease model. Thus, leptin-sensitive neuronal subpopulations in the LH enable adaptive fulfillment of vital needs despite anxiogenic conditions, both in healthy and pathological states.

## Abstract (web summary)

Figge-Schlensok et al. show that leptin-sensitive neurons in the lateral hypothalamus encode anxiogenic stimuli and facilitate adaptive strategies to enable an animal to overcome anxiety in threatening situations.

## Main

The interplay between emotional and homeostatic states can both enable and interfere with critical behavioral responses. For example, anxiety prevents exposure to harmful conditions and thereby fulfills the need for safety, but can also preclude essential behaviors such as feeding or exploration of territory. Adaptive strategies to overcome anxiety to fulfill a competing need are thus crucial for survival. Disruption of those adaptive strategies may underlie anxiety and eating disorders, two highly common mental health disorders that are often comorbid^1,2^. The mechanisms underlying the dynamic interaction between anxiety and eating are not understood. However, some evidence points to leptin as a factor involved in this interaction—the treatment with leptin, an adipocyte-derived hormone^3^, reduces anxiety in animal models^4^, as well as hyperactivity and depressive symptoms in anorexia nervosa patients^5^, and leptin receptor-expressing neurons in the lateral hypothalamus (LH; LepR^LH^) regulate competing basic needs such as eating, drinking and social exploration^6^. Here we combined single-cell Ca^2+^ imaging and cell-type-specific neuronal activity manipulations in those freely behaving mice, and identified neuronal circuit mechanisms that process anxiogenic conditions in sated and in hunger-induced anxiogenic states and counteract anxiety to facilitate adaptive behaviors.

## Results

### LepR^LH^ neurons counteract anxiety to enable exploration of exposed areas

**Fig. 1:**
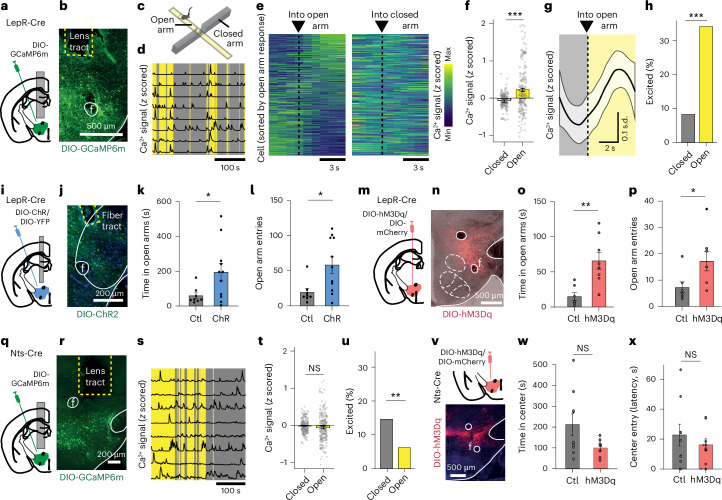
LepR^LH^ neurons counteract anxiety in the EPM. **a**–**h**, Single-cell Ca^2+^ imaging of LepR^LH^ neurons in freely moving mice (*n* = 193 cells, 16 mice, 10 females). **a**, GCaMP6m transduction and GRIN lens implantation above LH. **b**, Representative confocal image of expression and implantation. Scale bar = 500 μm. **c**, Schema of EPM. **d**, Representative Ca^2+^ transients. **e**, Heatmap of average responses around entry into open or closed arms, sorted by average response to open arm. Min, minimum; max, maximum. **f**, Average activity in open and closed arms (*P* = 7.2 × 10^−5^, paired Wilcoxon test). **g**, Activity dynamics of open arm-excited neurons around entry into open arm (local regression (LOESS) fit). **h**, Proportions of excited neurons (*P* = 1.1 × 10^−9^, two-sample *z* test). **i**–**l**, Optogenetic stimulation of LepR^LH^ neurons in the EPM (ctl, *n* = 8; ChR, *n* = 11 mice). **i**, ChR transduction and optic fiber implantation. **j**, Representative confocal image of ChR expression and fiber placement. Scale bar = 200 μm. **k**, Time in open arms (*P* = 0.0386, Student’s *t* test). **l**, Entries into open arms (*P* = 0.0157, Student’s *t* test). **m**–**p**, Chemogenetic activation of LepR^LH^ neurons in the EPM (ctl, *n* = 7; hM3Dq, *n* = 8 mice). **m**, hM3Dq transduction. **n**, Representative confocal image of hM3Dq expression. Scale bar = 500 μm. **o**, Time in open arms (*P* = 0.0028, Student’s *t* test). **p**, Entries into open arms (*P* = 0.0429, Student’s *t* test). **q**–**u**, Single-cell Ca^2+^ imaging of Nts^LH^ neurons in freely moving mice (*n* = 226 cells, 4 mice). **q**, GCaMP6m transduction and GRIN lens implantation above LH. **r**, Representative confocal image of expression and implantation. Scale bar = 200 μm. **s**, Representative Ca^2+^ transients. **t**, Average activity in closed and open arms (NS, paired Wilcoxon test). **u**, Proportions of excited neurons (*P* = 0.0055, two-sample *z* test). **v**–**x**, Chemogenetic activation of Nts^LH^ neurons in the OF (Ctl, *n* = 9; hM3Dq, *n* = 9 mice). **v**, hM3Dq transduction and representative confocal image of expression. Scale bar = 500 μm. **w**, Time in center zone (NS, Mann–Whitney *U* test). **x**, Latency to reach the center zone (NS, Mann–Whitney *U* test). Data represented as mean ± s.e.m., unless otherwise stated. *P < 0.05, **P < 0.01, ***P < 0.001. NS, not significant; f, fornix. Source data

**Extended Data Fig. 1:**
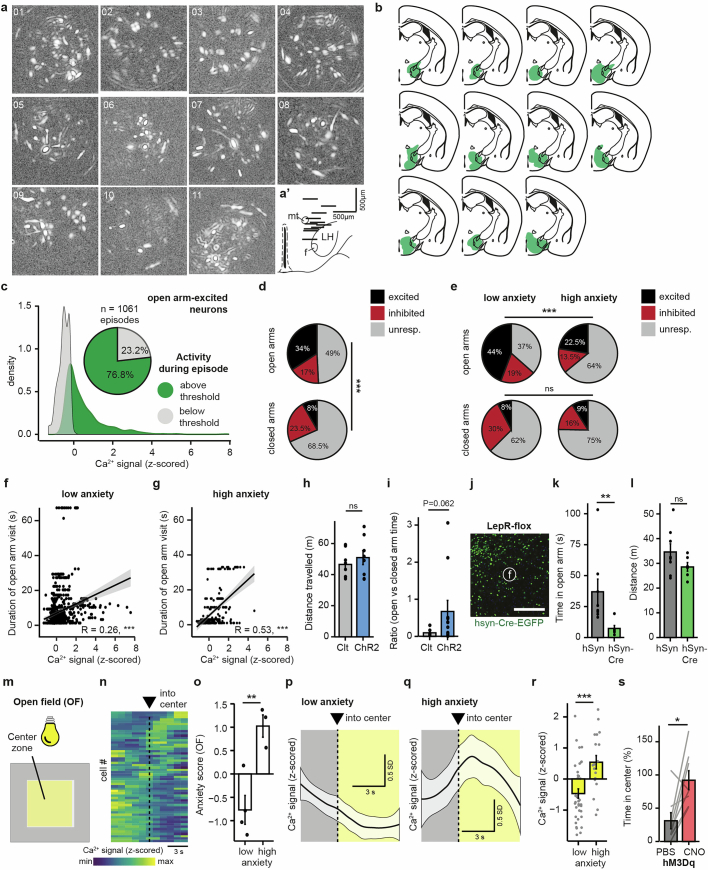
LepR^LH^ neurons encode and counteract anxiogenic stimuli in elevated plus maze and open field. **a**–**g**, Single-cell Ca^2+^ imaging of LepR^LH^ neurons in freely moving mice in EPM. **a**,**a’**, Representative projections of field of view (FOV) (**a**) and reconstruction of lens placement (**a’**). **b**, Evaluation of viral spread. **c**, Activity during episodes of open arm entries by open arm-excited LepR^LH^ neurons in the EPM paradigm. **d**, Proportions of response categories toward open- or closed arms (P = 3.9 ×10^−9^, X^2^ test). **e**, Proportions of response categories toward open- or closed arms in high- and low-anxiety animals (open arms: P = 0.0005, X^2^ test; closed arms: P = 0.0699, X^2^ test). **f**,**g**, Relationship between responses of open arm-excited neurons and duration of open arm visit in low-anxiety animals (**f**, R = 0.26, P = 1.9 ×1014) and high-anxiety animals (**g**, R = 0.53, P < 2.2 ×10^−16^, Pearson’s correlation; correlation in high- vs low-anxiety groups: P < 0.001, Fisher’s z transformation). **h**,**i**, Optogenetic stimulation of LepR^LH^ neurons in the EPM (ctl: n = 8, ChR: n = 11 mice). **h**, Distance traveled (ns, Student’s t-test). **i**, Ratio between time spent in the open and closed arm (P = 0.062, Mann–Whitney test). **j**–**l**, Cre-mediated ablation of LepR in LepR^LH^ neurons (ctl: n = 8, hsyn-Cre: n = 6 mice). **j**, Representative confocal image of Cre-mediated substitution of LepR with GFP in LepR^LH^ neurons. **k**, Time in open arms (P = 0.0027, Mann–Whitney U test). **l**, Distance traveled (ns, Mann–Whitney U test). **m**–**r**, Single-cell Ca^2+^ imaging of LepR^LH^ neurons in freely moving mice in open field test (n = 56 cells, 6 mice). **m**, Schema of open field test. **n**, Heatmap of LepR^LH^ activity around entry into center zone. **o**, Anxiety scores of low- and high-anxiety animals based on open field test (P = 0.0077, Student’s t-test). **p**,**q**, Activity dynamics around entry into the center zone in low-anxiety (**p**) and high-anxiety animals (**q**, LOESS fit). **r**, Average activity in center zone (P = 0.00084, Mann–Whitney U test). **s**, Chemogenetic activation of LepR^LH^ neurons: time in center (P = 0.015, paired t-test). Scale bars: **j**, 200 μm. Data represented as mean ± s.e.m., unless otherwise stated. ns—not significant, *P < 0.05, **P < 0.01, ***P < 0.001. Source data

To measure the responses of LepR^LH^ neurons to anxiogenic stimuli, we transfected LepR^LH^ neurons with the fluorescence indicator GCaMP6m and implanted a GRIN lens above the LH (Fig. 1a,b) to record the activity of individual LepR^LH^ neurons in mice that spontaneously explored the closed arms (safe zones) and open arms (exposed zones) of an elevated plus maze (EPM; Fig. 1c–e and Extended Data Fig. 1a,b). Across the whole population, LepR^LH^ neurons showed a higher average Ca^2+^ signal in the open arms compared to the closed arms (Fig. 1f), similarly in males and females (two-way analysis of variance (ANOVA): zone, *F*(1,382) = 36.86, *P* < 0.001; sex, *F*(1,382) = 0.84, NS; zone × sex, *F*(1,382) = 0.57, NS). Individual LepR^LH^ neurons showed elevated responses in the open arm (Fig. 1g and Extended Data Fig. 1c). The LepR^LH^ population contained a higher proportion of open arm-excited cells (34%) than closed arm-excited cells (8%; Fig. 1h and Extended Data Fig. 1d), and the proportion of open arm-excited cells was higher in low-anxiety animals than in high-anxiety animals (Extended Data Fig. 1e). Activity of open arm-excited LepR^LH^ cells correlated with time spent in the open arm, particularly in high-anxiety animals (correlation in high-anxiety versus low-anxiety groups, *P* < 0.001; Extended Data Fig. 1f,g).

To test the behavioral consequences of LepR^LH^ activation in anxiogenic environments, we performed complementary sets of neuronal activation experiments. First, we expressed the excitatory opsin channelrhodopsin 2 (ChR2) in LepR^LH^ neurons (Fig. 1i,j). Optogenetic activation of LepR^LH^ neurons increased both the time spent in (Fig. 1k) and the number of visits (Fig. 1l) to the open arms without affecting locomotion levels (Extended Data Fig. 1h,i), similarly in males and females (two-way ANOVA: male versus female, *F*(1,24) = 0.124, NS; group × sex, *F*(1,24) = 0.311, NS). Second, we expressed the excitatory designer receptors exclusively activated by designer drugs (DREADD) hM3Dq in LepR^LH^ neurons (Fig. 1m,n). This more prolonged, chemogenetic activation of LepR^LH^ neurons also increased both the time spent in the open arms (Fig. 1o) and the number of visits (Fig. 1p) to the open arms. Conversely, ablation of LepR from LepR^LH^ neurons—achieved by expressing AAV-hsyn-Cre virus in LH of LepR-floxed mice^7^—decreased the time animals spent in the open arms (Extended Data Fig. 1j–l).

To test whether the response of LepR^LH^ neurons to anxiogenic stimuli generalizes across tasks, we also measured LepR^LH^ responses toward the typically anxiogenic central zone of an open field (OF; Extended Data Fig. 1m,n). High-anxiety animals showed an increase in LepR^LH^ responses when approaching the center zone (Extended Data Fig. 1o–r). Chemogenetic activation of LepR^LH^ neurons in the OF increased the time spent exploring the center zone (Extended Data Fig. 1s).

These data suggest that activation of LepR^LH^ neurons by anxiogenic stimuli may counterbalance anxiety, enabling the exploration of new terrain. By contrast, the activity of neurotensin-expressing LH (Nts^LH^) neurons—another GABAergic LH population involved in the regulation of water and food intake^8^—did not differ between closed and open arms (Fig. 1q–t), and the proportion of individual Nts^LH^ neurons that were significantly excited in open arms was low (6%) and significantly lower than the closed arm-excited subgroup (14.6%; Fig. 1u). Moreover, chemogenetic activation of Nts^LH^ neurons did not affect anxiety-related behavior in the OF test (Fig. 1v–x), consistent with a previous study that did not detect changes in anxiety-related behavior in the EPM during Nts^LH^ activation^8^. Thus, activation by anxiogenic stimuli is specific to LepR^LH^ but not to Nts^LH^ neurons.

### Input from prefrontal cortex (PFC) inhibits LepR^LH^ neurons in high-anxiety animals

**Fig. 2:**
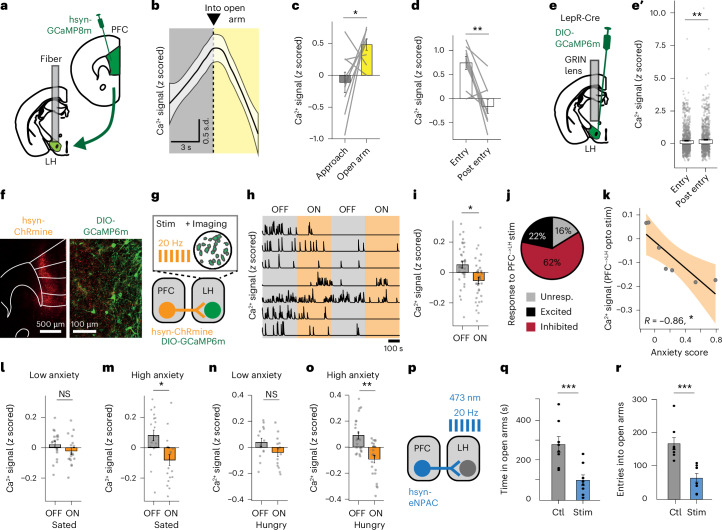
Input from PFC regulates the activity of LepR^LH^ neurons. **a**–**d**, Spontaneous activity of projections from PFC into LH (*n* = 9 mice). **a**, GCaMP8m transduction of PFC projections into LH and optic fiber implantation above LH. **b**, Activity dynamics of prefrontal inputs during spontaneous exploration of the EPM (LOESS fit). **c**, Activity before and after entry into the open arm (*P* = 0.039, paired *t* test). **d**, Activity during entry into the open arm and after entry (*P* = 0.0078, paired Wilcoxon test). **e**,**e’**, Average activity of open arm-excited LepR^LH^ neurons during entry into the open arm and after entry (**e’**, *P* = 0.0017, paired Wilcoxon test, *n* = 845 episodes). **f**–**o**, Simultaneous single-cell Ca^2+^ imaging of LepR^LH^ neurons and optogenetic stimulation of PFC^→LH^ inputs in sated (*n* = 37 cells) and hungry state (*n* = 36 cells) in high-anxiety and low-anxiety mice classified according to the extent of open arm exploration in EPM (*n* = 7 mice). **f**, Representative expression of ChRmine in PFC (left) and somatic expression of GCaMP6m in LepR^LH^ neurons alongside axonal expression of ChRmine in LH (right). Scale bars = 500 μm (left) and 100 μm (right). **g**, Simultaneous somatic Ca^2+^ imaging and axonal optogenetic excitation. **h**, Representative Ca^2+^ transients during optogenetic stimulation (ON) and between stimulation episodes (OFF). **i**–**m**, LepR^LH^ response to PFC^→LH^ input stimulation in sated state. **i**, LepR^LH^ response to PFC^→LH^ input stimulation (*P* = 0.02, paired *t* test, *n* = 73 cells). **j**, Proportions of responsive cells (inhibited, *n* = 23; excited, *n* = 8; unresponsive, *n* = 6). **k**, Relationship between PFC-mediated LepR^LH^ suppression and anxiety level exhibited in the EPM (*P* = 0.014, Pearson’s correlation). **l**,**m**, LepR^LH^ response to stimulation in sated state—low-anxiety animals (**l**; NS, paired *t* test, *n* = 18 cells) and high-anxiety animals (**m**; *P* = 0.034, paired *t* test, *n* = 19 cells). **n**,**o**, LepR^LH^ response to PFC^→LH^ input stimulation in hungry state—low-anxiety animals (**n**; NS, paired *t* test, *n* = 14 cells) and high-anxiety animals (**o**; *P* = 0.005, paired *t* test, *n* = 22 cells). **p**–**r**, Optogenetic stimulation of PFC^→LH^ inputs (*n* = 8 mice). **p**, Stimulation protocol. **q**, Time in open arms (*P* = 0.00039, paired *t* test). **r**, Entries into open arms (*P* = 1.1 × 10^−5^, paired *t* test). Data represented as mean ± s.e.m., unless otherwise stated. *P < 0.05, **P < 0.01, ***P < 0.001. Unresp., unresponsive; stim, stimulation. Source data

**Extended Data Fig. 2:**
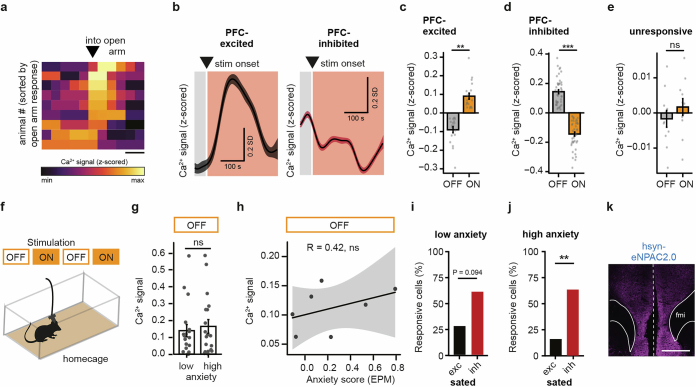
Regulation of LepR^LH^ cells by prefrontal cortex input and their baseline activity. **a**, Heatmap of PFC activity around entry into the open arm, scale bar: 2 s. **b**–**j**, Simultaneous single-cell Ca^2+^ imaging of LepR^LH^ neurons and optogenetic stimulation of PFC^→LH^ inputs in high- and low-anxiety mice classified according to extent of open arm exploration in EPM. **b**, Activity dynamics of PFC excited= (left) and PFC inhibited (right) cells upon onset of PFC^→LH^ input stimulation (GAM fit). **c**–**e**, Average LepR^LH^ response to PFC^→LH^ input stimulation in sated state in PFC-excited (**c**, P = 0.0046), PFC-inhibited (**d**, P = 3.1 ×10^−7^) and unresponsive (**e**, P = 0.37, paired t-test) LepR^LH^ neurons (n = 37 cells, 7 mice). **f**–**h**, Baseline LepR^LH^ activity in low- and high-anxiety animals (low-anxiety: n = 32 cells, high-anxiety: n = 41 cells). **f**, Stimulation protocol in the home cage. **g**, Activity during first OFF period (P = 0.78, Mann–Whitney U test). **h**, Relationship between baseline activity and anxiety score according to open arm exploration in the EPM (P = 0.35, Pearson’s correlation). **i**,**j**, Proportion of PFC-modulated neurons in sated state in low-anxiety animals (**i**, P = 0.094) and high-anxiety animals (**j**, P = 0.0079, two samples Z test). **k**, Representative expression of eNPAC2.0 in PFC, fmi = forceps minor of the corpus callosum, scale bar: 300 μm. Data represented as mean ± s.e.m., unless otherwise stated. ns - not significant, **P < 0.01, ***P < 0.001. Source data

The PFC conveys anxiety-modulating inputs^9,10^. The LH-projecting PFC neurons encode anxiogenic stimuli and behaviors, such as entry into a new environment^10^; however, their functional impact on hypothalamic subpopulations is unclear. To assess the contribution of PFC inputs to LH-mediated anxiolytic functions, we transfected PFC neurons with GCaMP8m, implanted an optic fiber into the LH and measured activity of PFC^→LH^ projections during free exploration of the EPM using fiber photometry (Fig. 2a). PFC^→LH^ inputs increased activity during approach of the open arm (Fig. 2b,c and Extended Data Fig. 2a) and rapidly decreased activity after entry into the open arm (Fig. 2d), whereas activity of LepR^LH^ neurons increased after entry into the open arm (Fig. 1g,h) and sustained high activity levels in the open arm (Fig. 2e,e’). These data indicate that prefrontal inputs to LH conveyed anxiety-related information and preceded anxiety-triggered activation of LepR^LH^ neurons.

To test whether LepR^LH^ neurons are indeed sensitive to PFC input, we expressed the red-shifted excitatory opsin ChRmine in PFC neurons and GCaMP6m in LepR^LH^ neurons (Fig. 2f) and recorded the activity of LepR^LH^ neurons during optogenetic stimulation of PFC projections in LH (Fig. 2g,h). Activity of the LepR^LH^ population decreased during PFC^→LH^ input stimulation (Fig. 2i), and the majority of LepR^LH^ neurons were classified as inhibited during activation of PFC input (Fig. 2j and Extended Data Fig. 2b–e). We then evaluated how prefrontal suppression of LepR^LH^ neurons relates to anxiety levels, according to the degree of open arm exploration in the EPM. At baseline, the activity of LepR^LH^ neurons was similar between low-anxiety and high-anxiety animals (Extended Data Fig. 2f–h). However, during stimulation of PFC^→LH^ inputs, the reduced activity of LepR^LH^ neurons positively correlated with anxiety levels (Fig. 2k). We then compared the effects of PFC^→LH^ input stimulation between animals that exhibited low-anxiety and high-anxiety levels in the EPM. Stimulation of PFC^→LH^ input did not have a net effect on LepR^LH^ activity in low-anxiety animals (Fig. 2l and Extended Data Fig. 2i). By contrast, in high-anxiety animals, stimulation of PFC^→LH^ input decreased LepR^LH^ activity, as the majority of the neurons were inhibited during stimulation (Fig. 2m and Extended Data Fig. 2j).

To test whether the sensitivity of LepR^LH^ neurons to PFC^→LH^ input stimulation persisted in the hungry state, we repeated the experiment after food restriction was imposed. In the hungry state, PFC^→LH^ input stimulation similarly inhibited LepR^LH^ neurons in high-anxiety animals but not in low-anxiety animals (Fig. 2n,o). To test if PFC^→LH^ input stimulation regulates anxiety-related behavior, we optogenetically activated PFC^→LH^ inputs during the exploration of the EPM (Fig. 2p and Extended Data Fig. 2k) and observed a decrease in both time spent in open arms and entries into open arms (Fig. 2q,r). Together, these findings suggest that prefrontal input into LH decreases the activity of anxiety-reducing LepR^LH^ neurons and increases anxiogenic responses.

### LepR^LH^ neurons counteract anxiety to promote feeding in an anxiogenic context

**Fig. 3:**
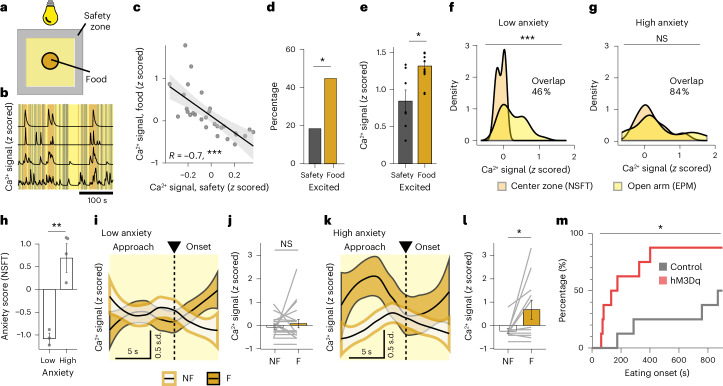
LepR^LH^ neurons counteract anxiety to promote feeding in an anxiogenic context. **a**–**l**, Single-cell Ca^2+^ imaging of LepR^LH^ neurons in NSFT after overnight food deprivation (*n* = 38 cells, 7 mice). **a**, Schema of NSFT. **b**, Representative Ca^2+^ transients. **c**, Relationship between food-elicited and safety-elicited responses (*R* = −0.7, *P* = 1.6 × 10^−5^, Pearson’s correlation, *n* = 29 cells, 6 mice). **d**, Proportion of cells excited in safety or food zone (*P* = 0.039, two-sample *z* test). **e**, Activity level across population in safety or food zone (*P* = 0.011, paired Wilcoxon test). **f**,**g**, Distribution of responses to anxiogenic spaces in EPM and NSFT in low-anxiety (**f**, *P* = 0.0005, Kolmogorov–Smirnov test) and high-anxiety animals (**g**, *P* = 0.8). **h**, Anxiety score of low-anxiety and high-anxiety animals classified according to latency of eating onset in NSFT (*P* = 0.0019, Student’s *t* test). **i**–**l**, Activity dynamics during food approach preceding NF (shaded in white) and successful F (shaded in gold). **i**,**j**, Low-anxiety animals—activity dynamics (**i**; generalized additive model (GAM) fit) and average activity level during approach (**j**; NS, paired Wilcoxon test). **k**,**l**, High-anxiety animals—activity dynamics (**k**; GAM fit) and average activity level during approach (**l**; *P* = 0.042, paired Wilcoxon test). **m**, Feeding performance in NSFT during LepR^LH^ activation (*P* = 0.0286, Mantel–Cox log-rank test; control, *n* = 7; hM3Dq, *n* = 8 mice). Data represented as mean ± s.e.m., unless otherwise stated. *P < 0.05, **P < 0.01, ***P < 0.001. NF, novelty-suppressed feeding; F, feeding. Source data

**Extended Data Fig. 3:**
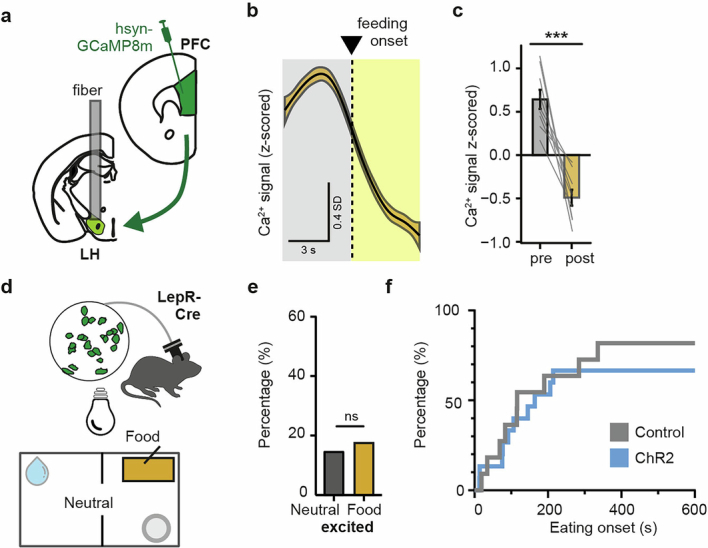
Activity of projections from prefrontal cortex into LH, and of LepR^LH^ cells, during feeding. **a**–**c**, Spontaneous activity of projections from prefrontal cortex into LH around feeding onset. **a**, Schema of fiber photometry recordings: GCaMP6m expression in PFC neurons and optic fiber implantation above LH. **b**, Activity dynamics of prefrontal inputs to LH around spontaneous feeding onset (LOESS fit). **c**, Activity before and after feeding onset (P = 0.00025, paired t-test). **d**,**e**, Single-cell Ca^2+^ imaging of LepR^LH^ neurons in familiar, dimly lit enclosure. **d**, Schema of free feeding enclosure (FFE). **e**, Proportion of neurons with significantly increased activity levels in stimulus-free (‘neutral’) zone or food zone (ns, two sample Z test; n = 166 neurons, 5 mice). **f**, Feeding performance in FFE during optogenetic LepR^LH^ activation (ns, log-rank (Mantel-Cox) test; control: n = 8, ChR2: n = 11 mice). Data represented as mean ± s.e.m., unless otherwise stated. ns—not significant, ***P < 0.001. Source data

High levels of anxiety, such as in an exposed, open space, would preclude eating unless an animal can rely on adaptive strategies to reduce anxiety levels around food. For instance, increased activity of LH-projecting PFC neurons during food approach prevents feeding, while inhibition of these neurons facilitates feeding in a novelty-suppressed feeding task (NSFT)^10^. In this test, in which food is placed in the center of a brightly lit, new arena, food-deprived animals experience a conflict between the drive to eat and anxiety (Fig. 3a). In agreement with this, we detected a strong increase in the activity of PFC^→LH^ projections during food approach and a rapid decrease when the animal was able to feed (Extended Data Fig. 3a–c). Thus, an increase in LepR^LH^ activity may functionally oppose prefrontal input, enabling feeding in an anxiogenic environment.

To test this hypothesis, we first measured the activity of LepR^LH^ neurons in the NSFT. Indeed, LepR^LH^ neurons strongly responded to the food stimulus in the central, anxiogenic location (Fig. 3b). Individual LepR^LH^ neurons tended to respond to either food or the safety zone, whereby food-elicited and safety-elicited responses of individual LepR^LH^ neurons were negatively correlated (Fig. 3c), and proportions and response amplitudes of food-excited neurons were higher compared to safety-excited neurons (Fig. 3d,e).

Next, we assessed whether the LepR^LH^ responses to anxiogenic stimuli differed between low-anxiety and high-anxiety animals (as classified based on time spent in the open arms in the EPM). In both groups, we compared LepR^LH^ responses to open arms of the EPM versus the center zone of the NSFT. In low-anxiety animals, the LepR^LH^ population differentiated between the two anxiogenic spaces (Fig. 3f), whereas in high-anxiety animals LepR^LH^ responses to the two anxiogenic stimuli largely overlapped (Fig. 3g).

To further assess anxiety-related responses of LepR^LH^ neurons in the NSFT, we classified animals as low-anxiety or high-anxiety animals based on the latency to feed in NSFT (Fig. 3h), and compared LepR^LH^ responses between episodes of novelty-suppressed feeding and successful feeding (Fig. 3i–l). In high-anxiety animals, eventual successful feeding was preceded by elevated LepR^LH^ activity during the approach to food (Fig. 3k,l). This was not observed in low-anxiety animals (Fig. 3i,j). The increase in LepR^LH^ activity before successful feeding in high-anxiety animals coincided with an increase in prefrontal activity (Extended Data Fig. 3a–c), suggesting that the LepR^LH^ activation counteracted inhibition from PFC to facilitate the onset of feeding under anxiogenic conditions. Indeed, in the NSFT, the suppression of feeding by novelty was reduced by chemogenetic activation of LepR^LH^ neurons, resulting in an earlier feeding onset (Fig. 3m). By contrast, in food-deprived mice in a nonanxiogenic environment—a familiar, dimly lit enclosure—the proportion of food-excited LepR^LH^ neurons was low, and optogenetic LepR^LH^ activation did not affect latency to feeding onset (Extended Data Fig. 3d–f). Thus, LepR^LH^ activation decreases the latency to eat specifically in an anxiogenic context.

### Anxiety levels are associated with expression of anorexia nervosa risk genes in LepR^LH^ neurons

**Fig. 4:**
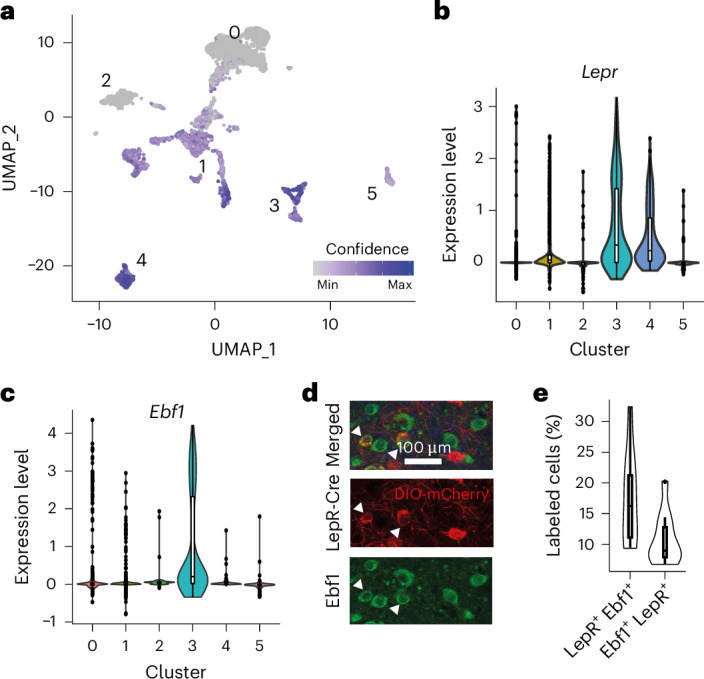
Expression of the anorexia nervosa-associated gene *Ebf1* in LepR^LH^ neurons. **a**–**c**, Transcriptional analysis of LH neurons. **a**, UMAP of *Lepr*^+^ and *Lepr-*LH clusters. Color code indicates confidence score for *Lepr* expression. **b**, Expression level of *Lepr*. **c**, Expression level of *Ebf1*. **d**,**e**, Analysis of LepR coexpression with Ebf1. **d**, Representative confocal image of LepR and Ebf1 coexpression in LH. Scale bar = 100 µm. **e**, Proportion of LepR^+^ cells (*n* = 1,560) coexpressing Ebf1 (left) and Ebf1^+^ cells (*n* = 2,428) coexpressing LepR (right), *n* = 12 hemispheres, 6 mice. **b**,**c**, Sample size—cluster 0, *n* = 2,290; cluster 1, *n* = 1,174; cluster 2, *n* = 347; cluster 3, *n* = 314; cluster 4, *n* = 231; cluster 5, *n* = 169 cells. **a**–**c**, Raw data available at National Center for Biotechnology Information Gene Expression Omnibus accession GSE188646 (ref. ^11^). Centerline in box indicates median, and top and bottom bounds of box indicate inter-quartile range (IQR) between first and third quartiles with whiskers indicating minima (−1.5× IQR) and maxima (+1.5× IQR). Source data

**Extended Data Fig. 4:**
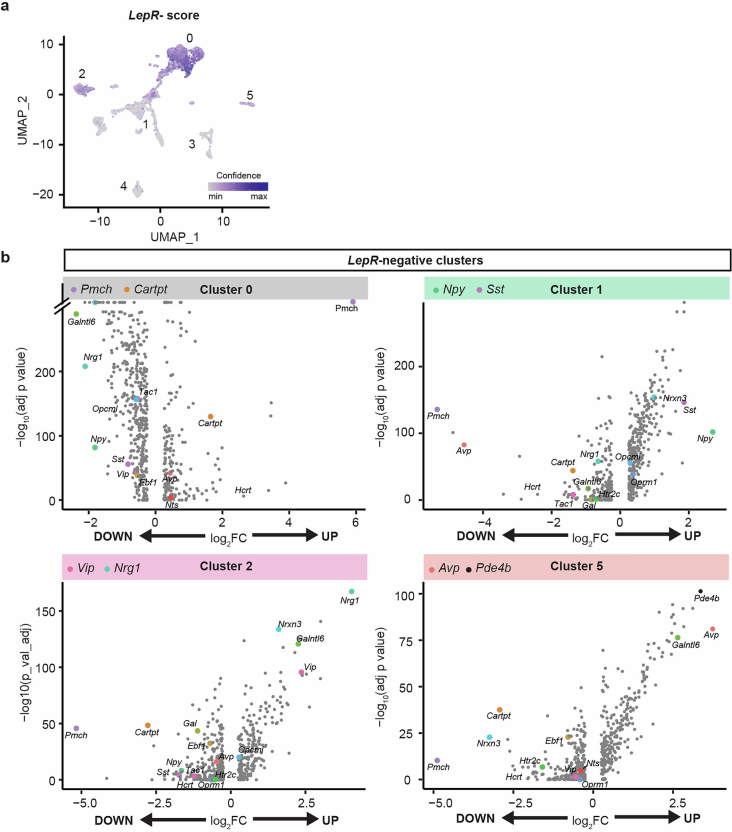
Gene expression in *Lepr*-negative clusters. **a**, UMAP of *Lepr*+ and *Lepr−* LH clusters, color code indicates confidence score for absence of *Lepr* expression. **b**, Volcano plots of gene expression in *Lepr−* clusters based on Wilcoxon rank sum test with Bonferroni multiple test correction. **a**,**b**, Raw data available at NCBI GEO accession GSE188646. Source data

**Extended Data Fig. 5:**
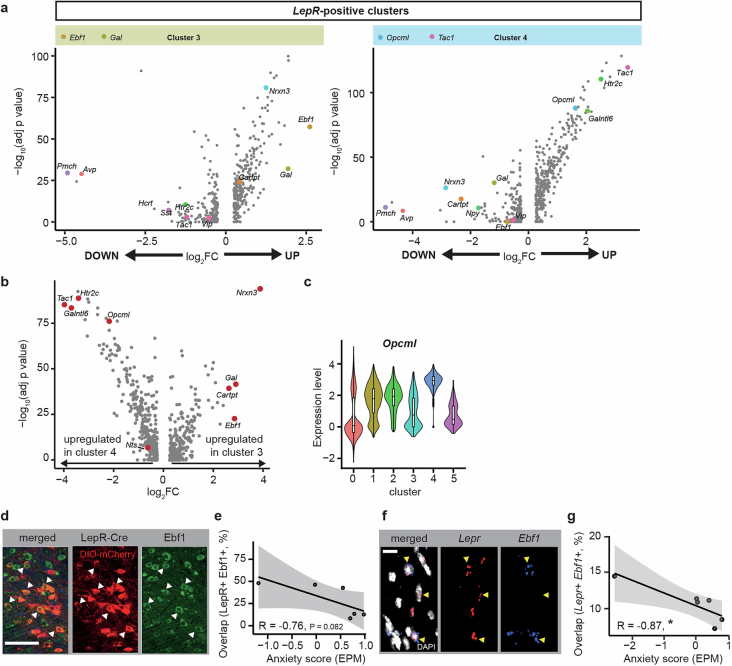
Gene expression in *Lepr*-positive clusters. **a**, Volcano plots of gene expression in *Lepr*+ clusters. **b**, Volcano plot of differential analysis of *Lepr*+ clusters 3 and 4. **c**, Expression level of *Opcml*. Sample size: cluster 0, n = 2290; cluster 1, n = 1174; cluster 2, n = 347; cluster 3, n = 314; cluster 4, n = 231; cluster 5, n = 169 cells. **d**, Representative confocal image of LepR+ Ebf1+ co-expression in LH, scale bar: 100 μm. **e**, Relationship between LepR+ Ebf1+ co-expression and anxiety score according to open arm exploration in the EPM (P = 0.082, Pearson’s correlation, n = 6 mice). **f**, Representative confocal image of *Lepr* and *Ebf1* co-staining using multi-labeling in situ hybridization, scale bar: 10 μm. **g**, Relationship between *Lepr* and *Ebf1* co-expression according to open arm exploration in the EPM (P = 0.029, Pearson’s correlation, n = 6 mice). **a**–**c**, Raw data available at NCBI GEO accession GSE188646. **a**,**b**, Volcano plots based on Wilcoxon rank sum test with Bonferroni multiple test correction. **c**, Centerline in box indicates median, upper and lower bounds of box indicate IQR between 1st and 3rd quartile with whiskers indicating minima (−1.5× IQR) and maxima (+1.5× IQR). Source data

Anxiety around eating and an inability to adaptively regulate anxiety responses and initiate eating behavior are hallmarks of restrictive eating disorders. To investigate whether the LepR^LH^ population expresses markers of anxiety disorders or restrictive eating disorders, we analyzed the transcriptomic profile of LepR^LH^ neurons in the female mouse LH, based on scRNA-sequence data GSE188646 (ref. ^11^). Thus, we focused on six clusters in total, with four clusters classified with high confidence as *Lepr*^−^ (Extended Data Fig. 4a) and two clusters classified with high confidence as *Lepr*^+^ (Fig. 4a,b and Extended Data Fig. 5a). The *Lepr*^−^ cluster 0 constituted the largest cluster and was characterized by high expression levels of *Mch* that encodes melanin-concentrating hormone, a neuropeptide involved in the regulation of feeding^12^ (Extended Data Fig. 4b). Cluster 0 also included the orexin/hypocretin (OX) population, an LH population known to signal hunger-induced stress responses that are alleviated by leptin treatment^13^. The *Lepr*^−^ cluster 1 expressed genes encoding neuropeptide Y (NPY) and somatostatin (SST) at high levels (Extended Data Fig. 4b), peptides involved in feeding regulation^14,15^. The *Lepr*^−^ cluster 2 expressed genes encoding vasoactive intestinal peptide and neuregulin 1 (Nrg1) at high levels (Extended Data Fig. 4b). Nrg1 is a trophic factor that binds to ErbB4, a receptor with anxiolytic properties in the amygdala^16^. The *Lepr*^−^ cluster 5 expressed *Pde4b* at high levels, which encodes phosphodiesterase-4 (Extended Data Fig. 4b), a known risk gene for post-traumatic stress disorder and anxiety^17^. Our differential expression analysis of the *Lepr*^+^ clusters indicated that cluster 3 is a part of the galanin-positive (Gal^+^) population (Extended Data Fig. 5b). The Gal^+^ LepR^LH^ population is a small, GABAergic population that makes up 12–15% of LH neurons^18^ and is involved in the leptin-sensitive regulation of food intake^19^. The other *Lepr*^+^ cluster 4 expressed genes encoding tachykinin 1 (Tac1) and the 5-hydroxytryptamine receptor 2 C (Htr2c) at high levels (Extended Data Fig. 5a,b). The Tac1^+^ LepR^LH^ population is a larger population that comprises approximately 30% of LH neurons^20^ and is sensitive to fasting^18^ and implicated in anxiety behavior^21^.

*LepR*^+^ cluster 3 expressed the gene encoding early B cell factor 1 (*Ebf1*) at high levels, while *Ebf1* expression was low in all other clusters (Fig. 4c–e and Extended Data Figs. 4b and 5a). *Ebf1* mutations are associated with anxiety disorders and anorexia nervosa^22,23^. The *Lepr*^+^ cluster 4 expressed *Opcml*, which encodes Opioid-binding protein/cell adhesion molecule, another anorexia nervosa risk gene^22,24^, whereas *Opcml* expression was low in all other clusters (Extended Data Figs. 4b and 5a–c).

To test whether Ebf1 coexpression with LepR in LH neurons is relevant for anxiety behavior, we transfected LepR^LH^ neurons with mCherry, measured anxiety behavior in both the EPM and the OF test and costained LepR^LH^ neurons for Ebf1 (Extended Data Fig. 5d). Low-anxiety animals as tested in the EPM tended to have larger LepR^+^ Ebf1^+^ populations (Extended Data Fig. 5e). We observed a similar negative correlation between *LepR*^+^ *Ebf1*^+^ coexpression (assessed using multilabeling in situ hybridization) and anxiety as tested in the EPM (Extended Data Fig. 5f,g). Together, our transcriptional analysis suggests that at least two, molecularly distinguishable, LepR^LH^ subpopulations have dissociable roles in the adaptive regulation of anxiety behavior, whereby Ebf1 expression in LepR subpopulation may be associated with reduced anxiety.

### LepR^LH^ neurons encode stages of anorexia nervosa disease model

**Fig. 5:**
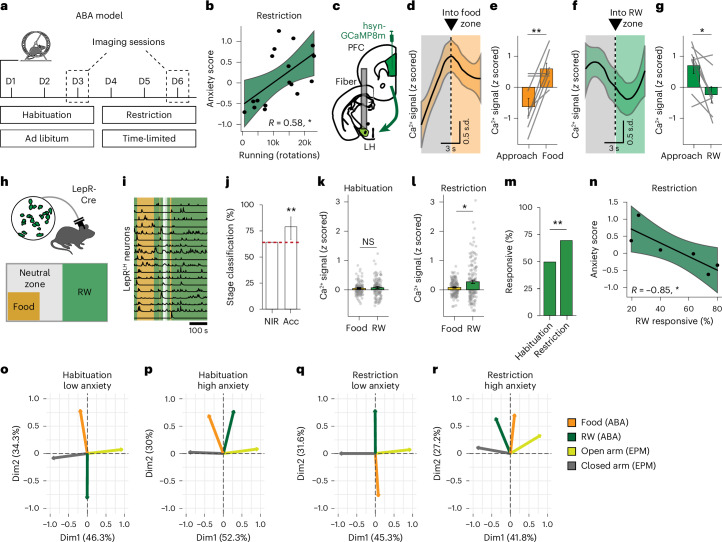
LepR^LH^ neurons encode stages of an anorexia nervosa disease model. **a**, Timeline of the ABA model. Imaging sessions were conducted during the time of scheduled feeding in the restriction phase, and the corresponding time window during the habituation stage. **b**, Relationship between overall anxiety score and running (*P* = 0.015, Pearson’s correlation). **c**–**g**, Spontaneous activity of projections from PFC into LH (*n* = 8 mice). **c**, GCaMP8m transduction of prefrontal inputs into LH and optic fiber implantation above LH. **d**, Activity dynamics around entry into the food zone during the restriction phase of the ABA. **e**, Activity before and after entry into the food zone (*P* = 0.0058, paired Wilcoxon test). **f**, Activity dynamics around entry into RW zone during the restriction phase of the ABA. **g**, Activity before and after entry into the RW zone (*P* = 0.02, paired Wilcoxon test). **h**–**n**, Single-cell Ca^2+^ imaging of LepR^LH^ neurons in ABA model (habituation, *n* = 109; restriction, *n* = 131 cells, 10 mice). **h**, Position of food and RW in ABA recording sessions. **i**, Representative Ca^2+^ transients of LepR^LH^ neurons during repeated entries into RW and food zone. Scale bar = 100 s. **j**, Classification of ABA stage based on LepR^LH^ activity in food, RW and neutral zone (NIR; Acc; *P* = 0.007, exact binomial test, error bars indicate 95% confidence intervals). **k**,**l**, Average LepR^LH^ response to food and RW during habituation (**k**; NS, paired Wilcoxon test) and restriction (**l**; *P* = 0.033, paired Wilcoxon test). **m**, Proportion of cells responsive to RW (*P* = 0.0026, *X*^2^ test) per ABA stage. **n**, Relationship between anxiety score and proportion of RW-responsive neurons (*R* = −0.85, *P* = 0.033, Pearson’s correlation). **o**–**r**, PCA of stimulus-evoked responses of LepR^LH^ neurons registered across ABA and EPM recordings in low-anxiety (**o**,**q**) and high-anxiety animals (**p**,**r**). Arrows indicate variable contributions in population space. Percentage values indicate variance explained per principal component. **d**–**f**, LOESS fit. Data represented as mean ± s.e.m., unless otherwise stated. *P < 0.05, **P < 0.01. NIR, no information rate; Acc, test accuracy. Source data

Anorexia nervosa is often comorbid with anxiety disorders^1,2^ and is characterized by reduced food intake and profound weight loss. A high proportion of patients exhibit increased, obsessive locomotor drive, which exacerbates weight loss. In animal models, activity-based anorexia (ABA) is a common and valid model for anorexia nervosa^25–27^. Here animals are provided with a running wheel (RW) in their home cage as well as ad libitum access to food (‘habituation’). After 3 days, food access is time-limited (‘restriction’; Fig. 5a). Similar to humans, hyperactivity in the ABA model—estimated by the activity in an RW—correlates with anxiety levels^28^ (Fig. 5b). Excessive locomotion is proposed to compensate or to relieve anxiety in humans^29^ and animal models^28^. Increased PFC activities during the presentation of food^30^ and anxiogenic stimuli^31^ are common features of eating disorders.

To characterize prefrontal responses to potentially anxiogenic stimuli in the ABA, we measured the activity of PFC^→LH^ inputs to food and RW in the ABA (Fig. 5c). We also observed a strong increase of activity during approach to and at contact with food (Fig. 5d,e), as well as high activity levels before the contact with an RW that rapidly decreased once the animals were inside the RW (Fig. 5f,g), indicating anxiogenic properties of food and anxiolytic properties of RW under ABA conditions.

**Extended Data Fig. 6:**
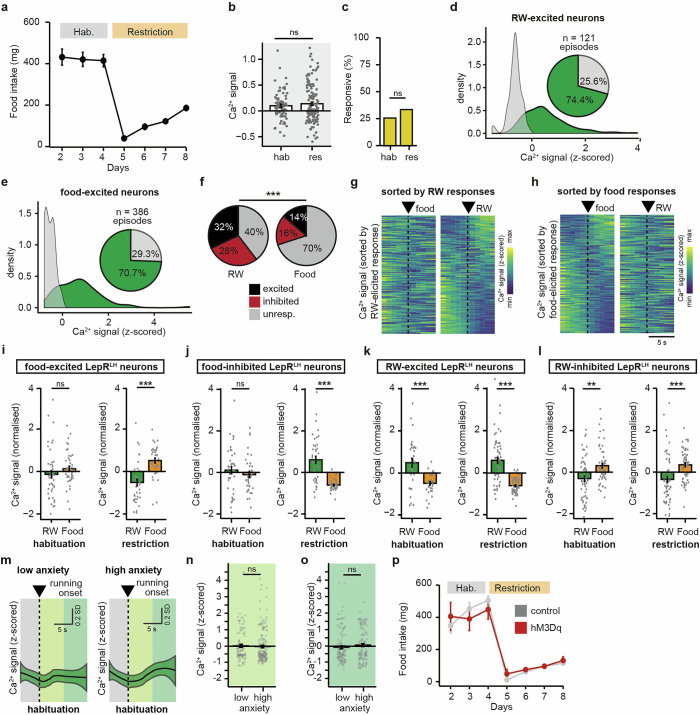
Activity of LepR^LH^ neurons in activity-based anorexia model. **a**–**o**, Single-cell Ca^2+^ imaging of LepR^LH^ neurons in the activity-based anorexia (ABA) model. **a**, Food intake throughout the ABA paradigm (n = 10 mice). **b**, Baseline activity in the stimulus-free, ‘neutral’ zone of the home cage used for ABA recordings (ns, Mann–Whitney U test, hab: n = 109, res: n = 131 cells, 10 animals). **c**, Proportion of cells responsive to food during habituation and restriction (ns, X^2^ test). **d**,**e**, Activity during episodes of RW (**d**) or food (**e**) location entries by RW- or food-excited (respectively) LepR^LH^ neurons in the ABA model. Episodes where activity exceeded threshold are indicated in green, episodes where activity did not exceed threshold are indicated in gray. **f**, Proportion of RW- or food-modulated LepR^LH^ neurons (P = 1.18 × 10^−10^, X^2^ test). **g**,**h**, Activity profile of LepR^LH^ neurons recorded during the habituation and restriction phase sorted according to amplitude of RW-elicited (**g**) or food-elicited responses (**h**). **i**–**l**, Responses to food or RW during habituation or restriction stage of food-excited (**i**), food-inhibited (**j**), RW-excited (**k**) or RW-inhibited (**l**) LepR^LH^ neurons (ns P ≥ 0.2, **P < 0.0028, ***P ≤ 1.4 ×10^−9^, paired Wilcoxon signed-rank test). **m**–**o**, Activity around onset of running during habituation phase (**m**, LOESS fit) and quantification in initial running phase (**n**, ns) and later running phase (**o**, ns, Mann–Whitney U test). **p**, Food intake during chemogenetic activation of LepR^LH^ neurons in the ABA paradigm (Ctl: n = 7, hM3Dq: n = 8 mice). Data represented as mean ± s.e.m. unless otherwise stated. ns—not significant, **P < 0.01, ***P < 0.001. Source data

To test whether the anxiolytic action of LepR^LH^ neurons promotes adaptive behavior during the progression of anorexia nervosa symptoms, we imaged the activity of LepR^LH^ neurons in female mice during both ABA stages (Fig. 5a; dashed squares) in the home cage during food access (Fig. 5h,i and Extended Data Fig. 6a). The activity of LepR^LH^ neurons predicted the habituation compared to restriction stages with high accuracy (79%; Fig. 5j). While baseline LepR^LH^ activity in the neutral zone outside of the food location or the RW was similar between habituation and restriction stage (Extended Data Fig. 6b,c), stimulus-evoked responses differed strongly—responses to RW increased during the restriction stage (Fig. 5k,l) and the proportion of RW-responsive cells increased during restriction compared to the habituation stage (Fig. 5m and Extended Data Fig. 6d–l). Moreover, animals with lower anxiety scores had a higher proportion of RW-responsive neurons (Fig. 5n), indicating that RW-elicited LepR^LH^ responses may reduce anxiety under ABA conditions.

To identify the main components that drive LepR^LH^ activity under anxiogenic conditions, we longitudinally registered the same LepR^LH^ neurons across both the phases of the ABA model and the EPM paradigm and performed principal component analysis (PCA). The LepR^LH^ population of low-anxiety animals exhibited well-separable stimulus-elicited trajectories (Fig. 5o,q), whereas trajectories were poorly distinguishable in population space of high-anxiety animals (Fig. 5p,r). Furthermore, the PCA analysis revealed strong state-specific differences—RW-elicited and food-elicited trajectories reversed in the population space of low-anxiety animals between habituation and restriction stages. Together, these data demonstrate strong modulation of RW-related LepR^LH^ responses by feeding state and anxiety levels.

### LepR^LH^ neurons enable adaptive behavioral responses in an anorexia nervosa disease model

**Fig. 6:**
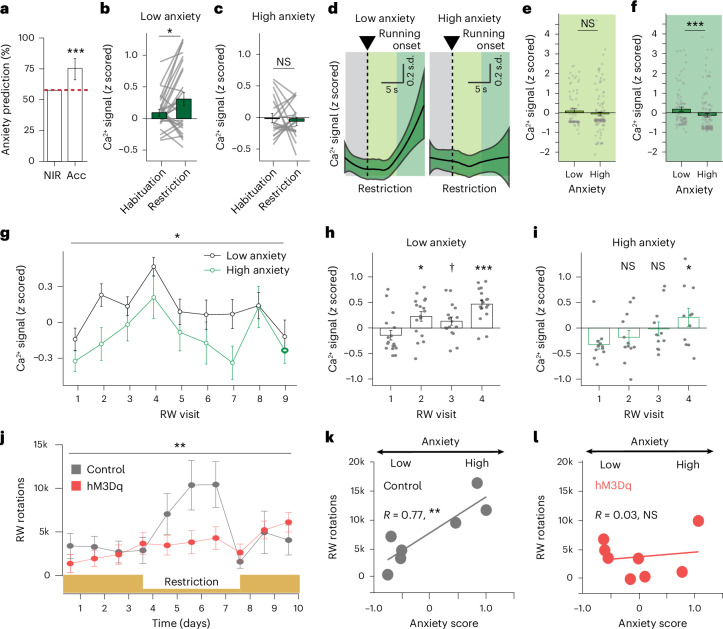
LepR^LH^ neurons enable adaptive behavioral responses in the ABA model. **a**–**i**, Single-cell Ca^2+^ imaging of LepR^LH^ neurons in ABA model. **a**, Decoding of anxiety category from RW-elicited LepR^LH^ activity (NIR; Acc; *P* = 9.18 10^−5^, exact binomial test, error bars indicate 95% confidence intervals). **b**,**c**, LepR^LH^ activity around RW entry during restriction in low-anxiety (**b**; *P* = 0.049, paired Wilcoxon test, *n* = 19 cells) and high-anxiety (**c**; NS, *n* = 27 cells) animals. **d**–**f**, Activity around onset of running during restriction phase (**d**; LOESS fit) and quantification in initial running phase (**e**; NS) and later running phase (**f**; *P* = 3.9 × 10^−5^, Mann–Whitney *U* test). **g**–**i**, RW-elicited activity in RW-excited LepR^LH^ neurons throughout sequential visits to RW in low-anxiety and high-anxiety animals. **g**, Activity across anxiety group-matched sequence of RW visits (*F*(8,128) = 2.289, *P* = 0.025, repeated measures ANOVA). **h**,**i**, Detailed quantification for initial set of RW visits (**h**, ^**†**^*P* = 0.057, *P = 0.0204, ***P = 2.2 × 10^−5^, *n* = 17 cells; **i**, *P = 0.033, Wilcoxon test with Bonferroni correction, *n* = 16 cells). **j**–**l**, Chemogenetic activation of LepR^LH^ neurons (ctl, *n* = 7; hM3Dq, *n* = 8 mice)—voluntary running levels (**j**; *F*(1.88, 23.19) = 7.337, *P* = 0.004, restricted maximum likelihood) and relationship between voluntary running and anxiety in control group (**k**; *P* = 0.009, Pearson correlation) and hM3Dq group (**l**; *P* = 0.721, Pearson correlation). Data represented as mean ± s.e.m., unless otherwise stated. *P < 0.05, **P < 0.01, ***P < 0.001. Source data

Our population analysis revealed strong anxiety-dependent and state-dependent differences in stimulus-evoked LepR^LH^ responses during ABA, suggesting that LepR^LH^ activity in the ABA reflects the anxiety level of the animal. Indeed, RW-elicited LepR^LH^ activity in the ABA predicted anxiety levels with high accuracy (76%; Fig. 6a). Furthermore, if LepR^LH^ activity contributes to the regulation of anxiety in the ABA, LepR^LH^ neurons of low-anxiety and high-anxiety animals should react differently to the anxiogenic (restriction) phase of the ABA. We tested this hypothesis using longitudinal registration of the same LepR^LH^ neurons across both habituation and restriction phases of the ABA.

LepR^LH^ neurons of low-anxiety animals (as defined by expression of anxiety behavior in the EPM) showed an increase in RW-elicited activity during the restriction phase in comparison to the habituation phase (Fig. 6b,c). This effect is due to a sharp increase in LepR^LH^ activity during running in the restriction phase, specifically in low-anxiety animals (Fig. 6d–f) that we did not observe in the habituation phase (Extended Data Fig. 6m–o). Furthermore, during the restriction phase, the activity of RW-excited LepR^LH^ neurons increased dynamically with sequential visits to the RW (Fig. 6g). In high-anxiety animals, RW-induced excitation developed slowly, resulting in an overall lower level of RW-elicited activation compared to low-anxiety animals (Fig. 6h,i).

These findings suggest that LepR^LH^ neurons in high-anxiety animals take longer to reach a sufficient level of activation through running to achieve an anxiolytic effect. This effect in high-anxiety animals may be due to the excessive PFC-mediated suppression of LepR^LH^ cells’ activity (Fig. 2m,o). In this case, increased LepR^LH^ activation may relieve anxiety and prevent maladaptive, compensatory locomotion during the restriction phase of ABA. To test this hypothesis, we chemogenetically activated LepR^LH^ neurons during all phases of ABA and found that locomotion during the restriction phase was reduced to baseline levels (Fig. 6j). Furthermore, chemogenetic LepR^LH^ activation uncoupled anxiety levels from the compensatory locomotion increase during the restriction phase, which was apparent in control animals (Fig. 6k,l and Extended Data Fig. 6p). Thus, anxiolytic LepR^LH^ activation may prevent excessive, compensatory locomotion under anorectic conditions and facilitate an adaptive response, that is, energy preservation.

## Discussion

Anxiety is an essential emotional state that helps to prevent exposure to harmful conditions. However, anxiety can also hinder the fulfillment of crucial needs, such as feeding, and may lead to maladaptive behaviors, such as excessive locomotion in anorexia nervosa. To balance these competing demands, animals and humans need to use adaptive strategies that allow them to overcome anxiety and meet essential needs despite all the anxiogenic conditions. The LH is a brain region essential for the integrative assessment of basic needs, such as feeding and drinking^32,33^. The treatment with leptin, an adipocyte-derived hormone^3^, reduces anxiety in animal models^4,34^ as well as hyperactivity and depressive symptoms in anorexia nervosa patients^5^. Here combining single-cell Ca^2+^ imaging and cell-type-specific neuronal activity manipulations in those freely behaving mice, we identified a leptin-sensitive population in the LH that enables adaptive behavioral responses under various anxiogenic conditions—exploration of exposed terrain to expand the radius of action space, eating despite an anxiogenic environment and limiting maladaptive excessive locomotion in an anorexia nervosa disease model.

While the constitutive disruption of LepR signaling leads to hyperphagia and obesity in humans and animal models^3^, and knockout of *Lepr* in LH increases food intake^35,36^, LepR^LH^-specific neural activity manipulations in animal models provide a more multifaceted perspective—whereas activation of LepR^LH^ neurons in sated animals in a familiar environment has little effect on food intake^6,37–39^, activation of LepR^LH^ neurons in hungry animals decreases food intake^6^, revealing state-dependent regulation of feeding behavior by LepR^LH^ neurons. Furthermore, subpopulations of LepR^LH^ neurons support different stages of feeding-related behavior, in particular food approach and consummatory phase^6,37,39^. High levels of anxiety, such as in an exposed, open space, would interfere with food approach and eventual consumption, unless an animal can rely on adaptive strategies to reduce anxiety levels around food. Here we found that LepR^LH^ neurons were activated by anxiogenic stimuli, including the open arms of an EPM and the center zone of an OF, as well as food when it was provided under anxiogenic conditions, such as in a new, brightly lit arena. While baseline LepR^LH^ activity was similar between low-anxiety and high-anxiety animals, as well as during sated and hungry states, the stimulus-evoked LepR^LH^ responses were highly distinguishable between states and anxiety levels—we observed different types of food responses during food approach in low-anxiety and high-anxiety animals, whereby an increase in LepR^LH^ activity during food approach predicted successful feeding under anxiogenic conditions in high-anxiety animals. Similarly, experimental activation of LepR^LH^ neurons facilitated feeding under anxiogenic conditions. However, LepR^LH^ activation did not affect feeding under baseline conditions. These results emphasize the integrative role of the LepR^LH^ population in regulating feeding behavior, accounting for both hunger pressure and anxiety level.

Maladaptive strategies to reduce anxiety may underlie symptoms of anxiety and eating disorders, which are highly comorbid^1,2^. While LepR^LH^ neurons in low-anxiety animals were able to clearly differentiate among anxiogenic stimuli, those in high-anxiety animals showed a reduced ability to make such distinctions, which could potentially impair adaptive behavioral responses and contribute to maladaptive behaviors. Anorexia nervosa patients often engage in excessive exercise to alleviate negative affective state^29^. Similarly, in animal models of anorexia nervosa, excessive exercise increases with anxiety level^27,28^. Here we detected strong differences between LepR^LH^ responses of low-anxiety and high-anxiety animals in an anorexia nervosa disease model—the ABA. While LepR^LH^ neurons did not respond to running during baseline (habituation phase of the ABA), and LepR^LH^ activation at this stage did not affect locomotion levels, LepR^LH^ neurons were activated by running during the restriction phase of the ABA. LepR^LH^ cells in high-anxiety animals took longer to activate during running, requiring more running to reach the same activation level as in low-anxiety animals. This delay may contribute to the excessive exercise associated with higher anxiety levels. Strikingly, induced activation of LepR^LH^ neurons precluded excessive locomotion during the restriction phase of the ABA model. Whereas LepR^LH^ activation does not affect locomotion levels in the EPM (regarding this study), or OF^40^, it increases locomotion in sated animals placed in a new home cage^38^ as well as in hungry animals actively searching for food^39^. Together, these data underscore the state-dependent, adaptive regulation of locomotion by LepR^LH^ neurons. While leptin levels are reduced in the ABA model^41^, we still observed responses to anxiogenic stimuli by LepR^LH^ neurons and anxiolytic effects during LepR^LH^ activation. One possible explanation may be increased leptin sensitivity observed during hypoleptinemia^42^. Furthermore, the activity of LepR^LH^ neurons may be regulated independently of leptin signaling through inputs from other anxiety-regulating brain regions (reviewed in ref. ^43^), such as bed nucleus of stria terminals (BNST)^44^, ventral hippocampus^45^ and PFC. The PFC conveys anxiety-modulating information^9,10^, and hyperactivity of the PFC is a biomarker for anxiety disorders^46^. Similarly, in eating disorders, increased PFC tone is typically observed during the presentation of food^30^ and anxiogenic stimuli^31^. Here we show that the PFC provides strong anxiogenic input to LH—prefrontal inputs rapidly responded to anxiogenic stimuli and activation of prefrontal inputs into LH increased anxiety behavior, whereas running on a wheel during food restriction in the anorexia nervosa disease model decreased activity levels of prefrontal inputs. Furthermore, activation of prefrontal input suppressed LepR^LH^ activity specifically in high-anxiety animals. The suppressive effects of prefrontal input onto LepR^LH^ neurons are likely polysynaptic, because prefrontal outputs are mostly glutamatergic^47,48^ (but see ref. ^49^). Together, these data suggest that high-anxiety levels are associated with greater inhibition of LepR^LH^ neurons by anxiogenic prefrontal input that prevents effective counteraction through LepR^LH^ activation. Aside from prefrontal input, LepR^LH^ neurons receive inhibitory inputs from anxiety-promoting subpopulations of the BNST^44^.

Neuronal populations in the LH are anatomically, molecularly and functionally distinct^32,33,50^. In this study, we described molecularly distinguishable subpopulations of *Lepr*^*−*^ and *Lepr*^+^ neurons. A large *Lepr*^*−*^ LH subpopulation (cluster 0) included the MCH^+^ population, an LH population involved in the regulation of feeding^12^ and the OX^+^ population, an LH population known to signal hunger-induced stress responses^51^. While OX activation is aversive and OX neurons receive negative valence inputs from lateral BNST, LepR^LH^ activation is appetitive and LepR^LH^ neurons receive positive valence inputs from medial BNST^44^, suggesting that LepR^LH^ neurons may functionally antagonize OX neurons. Indeed, hunger stress-induced hyperactivation of OX neurons is alleviated by the leptin treatment^13^, at least, in part, through LepR^LH^ neurons^13^, which inhibit OX neurons^52^. In contrast, Nts^LH^ neurons that lack LepR do not affect OX activity^53^, and Nts^LH^ neurons did not respond to anxiogenic stimuli (regarding this study). A smaller *Lepr*^*−*^ subpopulation (cluster 1) was marked by the expression of genes encoding NPY and SST. The glucose-sensing NPY^LH^ population is distinct from OX and MCH populations^54^. Intra-LH infusion of NPY is orexigenic^15^, while the effects of SST on food consumption are still under debate^14^. A third *Lepr*^*−*^ subpopulation (cluster 2) was marked by vasoactive intestinal peptide and Nrg1. Thus, Nrg1 is a trophic factor that binds to ErbB4 to regulate synaptic transmission and plasticity. Erbb4 is expressed widely throughout the brain^55^, modulating anxiety through amygdala and dorsal raphé circuits^16^. The fourth *Lepr*^*−*^ subpopulation (cluster 5) was marked by *Pde4b*, a known risk gene for post-traumatic stress disorder and anxiety^17^. Taken together, we identified *Lepr*^*−*^ subpopulations with a molecular profile implicated in anxiety, stress or depressive disorders, broadening the role of the LH in the regulation of anxiety-related behaviors.

We also identified the following two *Lepr*^+^ subpopulations: first, the *Lepr*^+^ subpopulation (cluster 3) is a part of the Gal^+^ population. The Gal^+^ LepR^LH^ population is a small, GABAergic population that comprises up to 12–15% of LH neurons^18^. Chemogenetic activation of the general Gal^LH^ population reduces anxiety^43,56^. While mutations of the *Gal* locus are associated with the severity of anxiety disorders in women^57^, systemic treatment with galanin improves anxiety behavior in some rodent assays^58^. Such anxiolytic effects of galanin may be due to the inhibitory effects of galanin on OX neurons in the LH^59^ or on noradrenergic neurons of locus coeruleus (LC)^60^, by either LC-projecting Gal^LH^ neurons^59^ or the local Gal^LC^ population^61^, or a combination of both. Chemogenetic activation of the general Gal^LH^ population increased operant food seeking in food-restricted animals^62^, suggesting that Gal^LH^ neurons can facilitate feeding, while we observed facilitation of feeding onset during LepR^LH^ activation specifically under anxiogenic conditions. Furthermore, voluntary exercise increases galanin expression in hypothalamus^63^ and LC^61^ and chemogenetic activation of the general Gal^LH^ population increases voluntary locomotion levels in sated animals^62^. We did not observe this effect during LepR^LH^ activation. Previous studies have also suggested the coexpression of Gal, Nts and corticotropin-releasing hormone (Crh) in a subset of LepR^LH^ cells^50^. However, Crh^LH^ activation increases feeding in the sated animal under nonanxiogenic conditions^64^, and we did not observe this effect during LepR^LH^ activation^6^. We also did not detect effects on anxiety-related behaviors during Nts^LH^ activation. Together, our data suggest that the anxiolytic role is specific to LepR^LH^ neurons, which may include an anxiety-responsive subset of the LepR^+^ Gal^+^ subpopulation, but not Nts^LH^ or Crh^LH^ populations. The *LepR*^+^ cluster 3 was further marked by expression of *Ebf1*. Ebf1 is critical for lipogenesis in a depot-specific manner, and knockout of Ebf1 results in low leptin levels^65^. Common genetic variants for *Ebf1* are associated with anxiety disorders and anorexia nervosa^22,23^. Here we found that high-anxiety animals were characterized by lower coexpression of Ebf1 in LepR^LH^ neurons.

Second, the *Lepr*^+^ subpopulation (cluster 4) is a part of the Tac1^+^ and Htr2c^+^ population. The Tac1^+^ LepR^LH^ population is a larger population^20,66^ that comprises approximately 30% LH neurons^18^ and is sensitive to fasting^18^. Tac1 signaling is implicated in anxiety behavior—mutation of *Tac1* (ref. ^21^) as well as pharmacological blockade of Tac1 signaling^67^ or mutation of the Tac1 receptor^68^ reduces anxiety levels and depressive-related behavior, potentially due to interactions with the serotonergic system^68^. Conversely, the treatment with Tac1 receptor agonists increases anxiety behavior^67^. While intracerebroventricular pharmacological manipulation of Tac1 receptor signaling does not affect locomotion in tests of anxiety and depression^67^, injection of substance P—a product of the *Tac1* gene—into brainstem regions^69^ or the mesolimbic system^70^ induces bouts of locomotion, although the endogenous sources of tachykinins are so far elusive. The *Lepr*^+^ (cluster 4) was marked by expression of *Opcml*, another known anorexia nervosa risk gene^22,24^. Further studies are needed to investigate the role of Ebf1 and Opcml in regulating LepR^LH^ excitability, particularly in response to anxiogenic cortical input.

Taken together, we identified leptin-sensitive neuronal subpopulations in the LH that encode anxiogenic stimuli and enable adaptive fulfillment of vital needs despite anxiogenic conditions, both in health and in a disease model. An exciting avenue for future studies would be to investigate the therapeutic utility of leptin-sensitive circuits for the treatment of anxiety and eating disorders, particularly in the context of genetic risk models.

## Methods

### Animals

Adult mice of LepR-Cre (JAX 032457) and Nts-Cre line (JAX 017525), as well as adult C57BL/6 (‘WT’) mice (JAX 000664), were bred in-house. Cre lines were backcrossed to the C57BL6/J strain. Mice of the age of 12 weeks were used for surgery. Mice reached approximately 16 weeks of age at the start of behavioral experiments. In all experiments, animals were randomly allocated to experimental groups. Heterozygous female LepR-Cre mice were used for single-cell microscopic calcium imaging and chemogenetic experiments. For calcium imaging experiments presented in Extended Data Fig. 3d,e, a cohort of male LepR-Cre mice was used. For calcium imaging experiments presented in Fig. 1q–u, imaging was performed in a cohort of adult male Nts-Cre mice. Optogenetic excitation of LepR-expressing neurons in the LH was conducted in a mixed cohort of LepR-Cre, and optogenetic excitation of prefrontal cortical projections was conducted in male WT mice. The cohort for fiber photometry experiments consisted of female WT mice. For a deletion of LepR-expressing cells in the LH, we used female mice of a LepR-floxed mouse line (LepR-flox; JAX, 008327). In situ hybridization was performed in female WT mice. Mice were kept under consistent room conditions of 22 °C (+/−2) and 55% (+/−5) humidity. After the surgery, mice were transferred to the experimental room with a reversed 12-h light/12-h dark cycle. All mice had ad libitum access to food (Ssniff, V1554-703) and water, unless stated otherwise. All experimental procedures were performed in accordance with national and international guidelines (ARRIVE) and approved by the local health authority (LANUV).

### Surgical procedures

Viral injections and implantation of GRIN lenses (0.5 numerical aperture (NA), 3/2 pitch, 0.6 × 7.3 mm; Inscopix) or 100-µm-diameter optical fibers (0.22 NA, Thorlabs), respectively, were performed in one surgery. Afterwards, mice were treated with carprofen (Rimadyl, Zoetis) for 3 days and then left for at least 3 weeks to ensure sufficient viral expression.

#### Viral injections

In preparation for the surgery, mice were injected with buprenorphine (0.1 mg kg^−1^; Buprenovet sine, Bayer) preceding the surgery. During the surgery, mice were kept under anesthesia using isoflurane (Baxter). Ointment (Bepanthen, Beyer) protected the eyes from drying. To ensure precise targeting, the head was fixated in a stereotaxic frame by Kopf Instruments (Tujunga). Mice were placed on a heating pad and a rectal thermometer was used to control body temperature throughout the surgery. To prepare the surgery site, fur was removed and lidocaine cream (EMLA, Aspen Pharma Trading) was applied to the skin. For the implantation of GRIN lenses, two screws (Bilany) were fastened to the skull to provide a better hold for the implant. A craniotomy was drilled with a trephine bit (1.8-mm diameter tip; Fine Science Tools) for virus injection, implantation and screws. Craniotomies were constantly cleaned with ice-cold PBS. Viral vectors (250 nl per level and 200 nl per level for chemogenetics cohort) were injected at a rate of 150 nl min^−1^, and 100 nl min^−1^ for fiber photometry experiments, respectively, using a stereotaxic-mounted nanopump (Hugo Sachs Elektronik—Harvard Apparatus) with a 30-gauge needle (Hamilton). The needle was halted at the injection site before the removal to allow for viral spread, and then was slowly removed. Mice of the calcium imaging, fiber photometry and optogenetic cohort were implanted subsequently. Animals of the chemogenetic cohort were sutured and treated with lidocaine cream (EMLA, Aspen Pharma Trading). Viral vectors were received from the UNC Gene Therapy Center Vector Core (University of North Carolina at Chapel Hill), Addgene (Watertown) or provided by the Deisseroth Lab (Stanford University). For calcium imaging experiments, AAVdj-Ef1a-DIO-GCaMP6m (GCaMP6m; UNC, 5 × 1,012 GC ml^−1^) was injected into LH of LepR-Cre mice. In addition, we injected AAV8-hSyn-mo20-33-oScarlet (hSyn-ChRmine, provided by K. Deisseroth, 4 × 1,012 vg ml^−1^) into the PFC for simultaneous axonal stimulation in LH. For somatic optogenetic stimulation of LepR neurons in LH (LepR^LH^), we injected AAV2-EF1α-DIO-hChR2(H134R)-EYFP (DIO-ChR2; UNC, 4 × 1,012 vg ml^−1^) into LH of LepR-Cre mice, and as a control we used AAV2-EF1α-DIO-EYFP (DIO-EYFP; UNC, 4.5 × 1,012 vg ml^−1^). Next, AAV8-hSyn-DIO-hM3D(Gq)-mCherry (DIO-hM3Dq; Addgene, 2.5 × 1,013 vg ml^−1^) was injected into LH of LepR-Cre and Nts-Cre mice for chemogenetic stimulation. AAV8-hSyn-DIO-mCherry (DIO-mCherry; Addgene, 2.1 × 10^13 ^vg ml^−1^) served as control. For axonal stimulation of LH-projecting PFC neurons, we injected AAVdj-hSyn-NpHR-TSp2A-hChR2(H134R)-eYFP (hSyn-eNPAC2.0, provided by K. Deisseroth, 1.84 × 10^12^ vg ml^−1^) or with AAV5-hSyn-EYFP (hSyn-EYFP; UNC, 3.3 × 10^12^ vg ml^−1^) into PFC of C57BL/6 mice. For fiber photometry experiments, AAV9-hSyn-jGCaMP8m-WPRE (Addgene, 2.65 × 10,132 vg ml^−1^) was bilaterally injected into the PFC of WT mice. LepR-flox mice were injected with AAV5-hSyn-HI-eGFP-Cre-WPRE-SV40 to achieve a targeted deletion of LepR-expressing cells in the LH. Control mice received the AAV5-hSyn-EGFP virus. Injection coordinates were determined relative to Bregma and according to the mouse brain atlas by Paxinos and Franklin (Paxinos & Franklin, 2001). The LH was targeted bilaterally at the following coordinates: anterior–posterior (AP), −1.3 mm; medial–lateral (ML), +/−0.9 mm and dorsal–ventral (DV, −5.2 mm and −5.4 mm. Bilateral injection sites for PFC were determined at the following coordinates: AP, 1.7 mm; ML, +/−0.35 mm; DV, −2.75 mm and −2.85 mm (for the fiber photometry cohort—AP, 1.8 mm; ML, +/−0.35 mm; and DV, −2.6 mm and −2.5 mm).

#### Implantation of GRIN lenses

We used GRIN lenses (0.5 NA, 3/2 pitch, 0.6 × 7.3 mm; Inscopix) with a diameter of 0.6 mm and a length of 7.3 mm to record calcium signal of LepR^LH^ neurons. After fixation of the lens holder (Pro-View kit, Inscopix), Bregma was redetermined at the tip of the lens. The lens was lowered at a rate of 100 µm min^−1^ until DV was −2.5 mm and at a rate of 50 µm min^−1^ until the targeted depth of DV was −5.0 mm relative to Bregma. Lenses were fixed using the C&B Metabond Quick Adhesive Cement System (Parkell). We used Parafilm, secured by a silicone elastomer (Kwik-Cast, World Precision Instruments), to protect the lens until the baseplate (Inscopix) could be attached approximately 3 weeks later. The baseplate ensured a correct and stable positioning of the miniscope during experiments. To place and fixate the baseplate, the mice were anesthetized again, according to the protocol described above. The miniscope of the calcium imaging system nVista (Inscopix) was attached to the stereotaxic frame with an adjustable gripper (Inscopix) to ensure correct placement and focus of the baseplate, as well as to check for calcium signal. Subsequently, the baseplate was fixated using C&B Metabond. Lenses were protected further by a protective cover (Inscopix).

#### Implantation of optic fibers

Optic fibers were custom built from optical fibers (0.22 NA; Thorlabs) with a length of approximately 5 mm. Fibers were connected to ceramic ferrules (Thorlabs) and secured by a two-component epoxy resin (R&G Faserverbundstoffe GmbH). After redetermining Bregma at the tip of the fiber, fibers were implanted bilaterally at the following coordinates: AP, −1.3 mm; ML, +/−1.0 mm and DV −4.9 mm; and at an angular position of AP, −1.3 mm; ML, +/−0.9 mm and DV, 4.9 mm, at 22 °C for somatic stimulation of LepR^LH^ neurons. For axonal stimulation of LH-projecting neurons from PFC, fibers were bilaterally implanted at the following coordinates: vertically at AP, 1.7 mm; ML, 1.0 mm and DV, −4.7 mm; and angularly at AP, 1.7 mm; ML, 1.0 mm and DV, −4.7 mm, at 21.8 °C. Finally, optic fibers were fixated with C&B Metabond.

#### Implantation of fiberoptic cannulas for fiber photometry

Borosilicate fiberoptic cannulas (400 μm, 0.66 NA; Doric Lenses) attached to a 1.25-mm-diameter metal ferrule were implanted in left LH (AP = −1.3 mm, ML = −0.9 mm and DV = −5.0 mm). The fiberoptic cannulas were fixated with C&B Metabond, and the implant was covered with a black nail polish.

### Single-cell microendoscopic calcium imaging

nVista and nVoke miniscopes, respectively, were attached through the baseplate. Upon removal of the protective cap, correct positioning was ensured through small magnets on the baseplate. In addition, the miniscopes were fixated with small screws.

#### Data acquisition and processing

Recordings of calcium signals were acquired at 10 frames per s with an average exposure time of 100 ms with Inscopix Data Acquisition Software. Focus of the miniscope was set individually for each mouse. Excitation-LED power was applied according to signal strength and varied between 0.7 mW mm^−^^2^ and 1.3 mW mm^−^^2^. For simultaneous optogenetic stimulation of axonal terminals projecting from PFC with the nVoke system, the optogenetic LED was set to 1.5 mW mm^−^^2^, and switched between ‘off’ and ‘on’ in a 5-min cycle. Synchronicity between recordings of calcium signals and behavioral tracking was ensured by sending transistor–transistor logic (TTL) pulses through an AMi-2 Optogenetic interface (Stoelting). Recordings were initialized with the behavioral tracking software ANY-maze (v7.14; Stoelting) and behavioral videos were recorded using monochrome cameras (30 Hz frame rate; Imaging Source Europe GmbH, DMK22BUC03). The Inscopix Data Processing Software was used for preprocessing in the form of spatial downsampling, spatial bandpass filtering and motion correction. Subsequent cell detection and trace extraction were done in a modified MATLAB (MathWorks, version R2017b) script, based on the CNMF-E library for MATLAB^71^ (https://github.com/zhoupc/CNMF_E/tree/master). Detected cells were manually curated to exclude components with traces that contained motion artifacts, high noise levels or those not displaying a round, soma-like shape. For longitudinal registration, a combination of automated registration with MATLAB-based CellReg GUI (https://github.com/zivlab/CellReg/tree/master) as well as manual registration was used.

### Optogenetic stimulation and acquisition

Optic fiber implants were checked before the implantation to ensure light delivery of at least 50%. Implants were connected to a 3-m fiberoptic patch cord with protective tubing and an FC/PC adaptor (25-mm diameter, 0.1 NA; Thorlabs) via a ceramic mating sleeve (1.25-mm diameter; Thorlabs). For somatic stimulation of LepR^LH^ neurons, as well as for stimulation of axonal terminals projecting from PFC to LH, a 473-nm diode-pumped solid-state laser (Laserglow Technologies, R471005FX) was triggered using TTL pulses, generated by an AMi optogenetic interface (Stoelting) and initiated by a protocol set in the behavioral tracking software ANY-maze (v6.33; Stoelting). Unilateral or bilateral stimulation was set to 5-ms pulses at 20 Hz. Laser output at the tip of the patch cord was measured with a power meter (PM100D, Thorlabs) to ensure a consistent outcome of approximately 22 mW. Behavioral videos were recorded using monochrome cameras (30-Hz frame rate, DMK22BUC03, Imaging Source Europe GmbH) controlled by ANY-maze (v6.33, Stoelting).

### Chemogenetic stimulation and acquisition

For chemogenetic stimulation of LepR^LH^ or Nts^LH^ neurons, mice expressing DIO-hM3Dq and DIO-mCherry, respectively, were injected with clozapine-N-oxide (CNO; 1 mg kg^−1^ i.p.; Hellobio) 60 min (LepR^LH^) or 30 min (Nts^LH^) before the start of the experiments. In the case of the ABA model, mice were injected daily, 1 h before the beginning of the dark phase. Behavioral tests were recorded with monochrome cameras (30-Hz frame rate; Imaging Source Europe GmbH, DMK22BUC03) and behavior was tracked with ANY-maze (v7.14; Stoelting).

### Fiber photometry recording

Fiber photometry experiments were performed with a Fiber Photometry System (Doric Lenses) and controlled by Doric Neuroscience Studio V6 software (v6.2.4.0; Doric Lenses). GCaMP8m was excited using 405 nm (~15 µW) and 465 nm (~30 µW) LED light driven by an LED driver (Doric Lenses), which was sinusoidally modulated at 572.205 Hz and 208.616 Hz. The light was then bandpass filtered at 400–410 nm and 460–490 nm. The 405-nm LED was used as isobestic wavelength for GCaMP8m.

#### Data acquisition and processing

Fiberoptic patch cords (400-µm core, NA 0.57, Doric Lenses) provided a light path between the parts of the system and the animals. Zirconia sleeves (Doric Lenses) were used to connect a fiberoptic patch cord to the implanted fiber. The emission light passed through the same optic fiber was bandpass filtered at 500–540 nm, collected by a built-in photodetector system (Doric Lenses) and demodulated. The DC detection mode was used, and the amplification level (1× or 10×) was adjusted for each recording to avoid signal saturation. The demodulated signal was downsampled and saved at 30 Hz. Behavioral videos were recorded with a monochrome camera (Basler ace, acA1920-40 µm) with an infrared illuminator (Stoelting) at 30 Hz. The camera was controlled by Pylon Viewer software (Basler AG). Video recordings were triggered by TTL signals generated at the Fiber Photometry Console (Doric Lenses), and aligned with the fiber photometry recordings. The first 5 s of the fiber photometry recordings were cut to eliminate noise associated with startup transients. Packet loss artifacts were identified as values exceeding 4× IQR within a 20-s sliding window and replaced by linear interpolation. The signals were then smoothed using a moving average filter with a sliding window of 1 s. The baseline was fitted with a double exponential function and subtracted from the signal to account for bleaching. Baseline correction was performed separately for the control and primary channels. The isobestic control channel was then fitted to the primary channel using linear regression; however, the fitted control was subtracted from the primary channel for motion correction. Δ*F*/*F* was calculated as the corrected signal divided by the previously fitted double exponential baseline. Corrected signal traces were *z* scored across the whole recording session.

### Behavioral assays

#### ABA model

The ABA model was conducted in home cages. RWs (TSE Systems) were mounted on custom-built three-dimensional-printed holders. Wheels were connected through AMi-2 digital interface (Stoelting) to allow continuous monitoring of RW activity by ANY-maze (v7.14; Stoelting). The protocol was based on the established ABA model described in ref. ^72^. The protocol consisted of three phases. In the habituation phase, mice had ad libitum access to food and got accustomed to the RW. During the restriction phase, access to food was restricted to 1.5 h, beginning with the dark phase (ZT12). The RW was always accessible. In the recovery phase, mice were given ad libitum access to food while maintaining access to the RW. Calcium imaging sessions were scheduled for the end of each phase to ensure proper habituation or restriction, respectively. These sessions took place at the beginning of the dark phase. Food was provided in a 10-cm Petri dish (Falcon) and food intake was determined with a fine scale (Sartorius AG). Mice had ad libitum access to water. Tracking data of calcium imaging sessions were received from ANY-maze (v7.14, Stoelting) and videos were recorded with a monochrome camera (30-Hz frame rate; Imaging Source Europe GmbH, DMK22BUC03). In addition to automated tracking by ANY-maze, videos were manually scored using BORIS^73^ (v8.21.8). In chemogenetic experiments, controlled and experimental animals received CNO (i.p., 1 mg kg^−1^) 1 h before the onset of the dark phase.

#### EPM

The EPM was a custom-built arena, consisting of two elevated crossing walkways. Two opposing walkways were enclosed by walls with a height of 15 cm, referred to as ‘closed arms’, while the other two walkways did not have walls, referred to as ‘open arms’. Each walkway measured 30 × 5 cm, and the walkways were elevated to 35-cm height. The test lasted for 15 min. The tests were performed during the dark phase. Mice were initially placed in the center of the arena. For optogenetic testing, laser stimulation was present throughout the test. For chemogenetic stimulation, mice were injected with CNO 1 h before the test. For optogenetic stimulation of LH-projecting PFC neurons, we used sham stimulation as an additional internal control. After each mouse, the platform was cleaned with disinfectant (Bacillol). Tracking data were received from ANY-maze (v7.14; Stoelting) and videos were recorded with a monochrome camera (30 Hz frame rate; Imaging Source Europe GmbH, DMK22BUC03).

#### OF test

For the OF test, we used a square arena (width × depth × height (WDH) = 45 × 45 × 40 cm), which was brightly lit (400–500 lux). The tests were performed during the dark phase. Each test lasted 15 min and started upon placing mice in one corner of the arena facing the walls. After each mouse, the platform was cleaned with disinfectant (Bacillol). Tracking data were received from ANY-maze (v7.14; Stoelting) and videos were recorded with a monochrome camera (30-Hz frame rate; Imaging Source Europe GmbH, DMK22BUC03). For Nts^LH^ animals, each test lasted for 20 min.

#### NSFT

NSFT took place in a new arena. The arena measured WDH = 45 × 45 × 40 cm with an uneven floor. Food was placed in the middle of the arena in a plastic lid (50-ml Falcon tube), and fixed with adhesive pads (UHU Patafix). Light conditions were increased to 498–514 lux. Preceding the test, mice were food-deprived for 22 h. The tests were performed during the dark phase. To initiate the test, mice were placed in the corner facing the arena walls. The protocol was started upon placing mice in the arena. Feeding and sniffing bouts were tracked manually during the test. Mice for chemogenetic stimulation were injected with CNO 1 h before the test. After each mouse, the platform was cleaned with disinfectant (Bacillol). Tracking data were received from ANY-maze (v7.14; Stoelting) and videos were recorded with a monochrome camera (30-Hz frame rate; Imaging Source Europe GmbH, DMK22BUC03).

#### Home cage recordings

To investigate changes in LepR^LH^ activity upon stimulation of PFC axonal terminals, the nVoke miniscope was attached (Inscopix) and mice were kept in their home cages for free exploration. The stimulation protocol was set to a 5-min OFF/ON cycle and the whole test lasted for 20 min. Recordings took place under both satiated and hungry conditions. For the latter, mice were food restricted for 4 days. Before food restriction, we predetermined the average body weight and the amount of food individual animals consumed each day, and restricted their normal daily intake for 4 days such that animals lost weight continuously but not more than 20% of their initial body weight^8^. Tracking data were received from ANY-maze (v7.14; Stoelting) and videos were recorded with a monochrome camera (30-Hz frame rate; Imaging Source Europe GmbH, DMK22BUC03).

#### Free feeding enclosure

For optogenetic stimulation of LepR^LH^, the free feeding enclosure was used as described previously^6^. In brief, in a rectangular arena (WDH = 50 × 30 × 40 cm), we presented the following four different stimuli: food, water, an object and a social stimulus. Preceding the tests, mice were familiarized with the arena; however, the social stimulus was always new. After 22 h of food deprivation, mice were allowed to explore freely in the arena under constant optogenetic stimulation.

#### Calculation of anxiety scores

For anxiety tests, such as the EPM and the NSFT, one main value for each test is used to assess the anxiety-related behavior of mice. These were as follows: time spent in the open arms (EPM), latency to eat (NSFT) or time spent in the center of the arena (OF). To evaluate anxiety levels of mice across tests, we normalized these values. For each of the tests, individual values were subtracted by the group average and the results were then divided by the group’s s.d. Anxiety scores of the EPM and the OF test were multiplied by −1. In short, the higher the anxiety score, the more anxious behavior was displayed by the mouse. We also calculated a general anxiety score by averaging anxiety scores of EPM, NSFT and OF test. In each anxiety test (that is, EPM, NSFT, OF test) and similar to previous studies^74^, we classified as ‘low-anxiety’ those animals with an anxiety lower than the average of the whole cohort tested and, conversely, as ‘high-anxiety’ those animals with an anxiety score higher than the average of the cohort. To ensure that our definition would apply to both normal and non-normal distributions of anxiety scores across a cohort, we used the median anxiety score of the cohort.

### Histology

Shortly after the last behavioral tests, mice were terminally anesthetized and perfused. Brains were further stored in 4% paraformaldehyde (Sigma-Aldrich) for coronal vibratome sectioning. Correct implant position and successful virus expression were confirmed using either a confocal microscope (Leica Camera AG, Leica SP8) or a wide-field microscope (Thermo Fisher Scientific, EVOS FL Auto 2 Imaging System), which enabled the analysis of fluorescence markers by comparing them to a standardized atlas of the mouse brain^75^.

#### Immunohistochemistry

We used brain slices of the chemogenetic control group of LepR-Cre mice, which expressed mCherry specifically in LepR^LH^ cells, for antibody stainings against Ebf1. Hypothalamic slices were transferred to 1× citrate buffer and placed on the thermoshaker at 80 °C for 30 min for antigen retrieval and were blocked consecutively with goat serum for 1 h at room temperature. For immunohistological stainings against Ebf1 (Rb; Sigma-Aldrich, AB10523; 1:200), slices were incubated with primary antibodies at 4 °C for 18 h on a shaker. Afterwards, we incubated the slices with antirabbit Alexa-Fluor 488 (Abcam, AB150077; 1:500) secondary antibody for 2 h. Before and after each incubation step, slices were washed in PBS.

#### Multilabeling fluorescence in situ hybridization

Perfused brains were stored in 4% paraformaldehyde (Sigma-Aldrich) for 1 h and then transferred to 30% sucrose (Merck) dissolved in 1× PBS (Carl Roth) overnight at 4 °C for cryoprotection. Brains were transferred to an embedding mold (Polyscience, Square S-22), embedded in Tissue Freezing Medium (Leica) on dry ice and sectioned at 14-µm thickness (Leica, CM3050S). Slices were dried at room temperature for at least 48 h. Multilabeling fluorescence in situ hybridization was performed using the RNAscope Multiplex Fluorescent Reagent Kit v2 (Advanced Cell Diagnostics, 323110) using a mix of the following two probes: C1, Mm-Ebf1 (Mus musculus Ebf1 transcript variant 1 mRNA, 433411), with TSA Vivid Fluorophore 650 (PN323273), and C3, Mm-Lepr (Mus musculus leptin receptor (ob-r) mRNA complete cds, 402731), with TSA Vivid Fluorophore 570 (PN323272). One slide (containing six to eight slices) for each animal was washed in autoclaved 1× PBS. PBS was replaced by 75% and then 100% ethanol (Merck Chemicals GmbH). Slides were dried for approximately 5 min (until dry), outlined with a PAP Pen, and incubated in hydrogen peroxide (H_2_O_2_) for 10 min at room temperature, followed by two rinses in 1× PBS. Protease III was applied, and slides were incubated at 40 °C for 30 min, then rinsed twice with 1× PBS. The probe mix (undiluted for C1 probe; 1:50 dilution for C3 probe; approximately 200-µl per slide) was added and incubated at 40 °C for 2 h. Slides were then rinsed twice with wash buffer (1:50 dilution in Milli-Q water) for 2 min each. Amplification steps were performed sequentially at 40 °C, each followed by twice 2 min rinses in wash buffer at room temperature—AMP1 and AMP2 incubation was applied for 30 min and AMP3 incubation was applied for 15 min. In addition, HRP-C1 was added and incubated for 15 min, followed by twice 2 min rinses in wash buffer. The Opal dye mix for C1 (Vivid Fluorophore 650, 1:750 dilution in TSA buffer) was applied and incubated for 30 min, followed by twice 2 min rinses in wash buffer. The HRP blocker was then applied for 15 min, followed by two additional rinses in wash buffer for 2 min each. Subsequent rounds of amplification were performed for channel C3. HRP-C3 was applied for 15 min, followed by twice 2 min rinses in wash buffer. The Opal dye mix for C3 (Vivid Fluorophore 570, 1:750 dilution in TSA buffer) was added and incubated for 30 min, followed by a 2-min wash in wash buffer. HRP blocker was then applied for 15 min, followed by twice 2 min rinses in wash buffer. Sections were counterstained with DAPI for 30 s and mounted using Aqua-Poly/Mount (Polyscience).

#### Quantification

Confocal images were taken with a Leica SP8 (Leica Camera AG) using the Leica Application Suite X. Identification of LepR^+^ and Ebf1^+^ cells was performed in Fiji, as discussed in ref. ^76^. For the anatomical definition of the LH, the internal capsule and the fornix were used as landmarks. mCherry-expressing cells were automatically defined with CellPose (v3.0.10). Cells labeled using immunohistochemistry as well as in situ hybridization were counted using the Cell Counter in Fiji.

### Data analysis

#### Identification of stimulus response profile

First, we binned Ca^2+^ signals (1 s bins) across the session, marked the occupation of each stimulus zone with ones when it occurred and identified entry into and exits from a stimulus zone. To visualize peri-stimulus activity across cells, we applied local polynomial regression fitting by weighted least squares (‘stats’ package, R) or, for large datasets, general additive models with smoothness estimation (‘mgcv’ package, R) over Ca^2+^ signals across time points per epoch starting at −5-s pre-entry and ending at 15-s postentry. To estimate stimulus-evoked neuronal activity per cell, we used the pre-entry epoch in a 3-s window before entry and the postentry epoch in a 3-s window after entry of the stimulus location. We applied a generalized additive model with penalized thin-plate regression splines (‘mgcv’ package, R) over Ca^2+^ signals of individual neurons across time points per epoch. For the NSFT, to visualize neuronal responses during novelty-suppressed and successful feeding, we applied local fitting per epoch starting at −10 s before food contact and ending at 5 s after food contact. To quantify neuronal responses leading up to novelty-suppressed and successful feeding, we averaged activity during food location approach per cell in a 3-s window starting at −6 s and ending at −3 s before food contact. To compare responses during open arm entry with responses after open arm entry, we used the peri-entry epoch in a 3-s window of −1 s before entry until 1 s after entry, and a postentry epoch of a 3-s window from 2 s to 4 s after entry. To compare responses to running, we also used the postentry epoch in a 3-s window from 4 s to 6 s after entry in addition to the initial 3-s window from 0 s to 3 s after entry.

#### Classification of stimulus-responsive cells

We identified stimulus-excited and stimulus-inhibited cells that exhibited systematic increases or decreases in Ca^2+^ activity when the animal occupied a stimulus location. We applied a linear model (‘stats’ package, R) to fit the Ca^2+^ signal of individual cells. For the ABA task, the following predictors were used: occupation of food zone, RW zone and neutral zone. For the NSFT task, the following predictors were used: occupation of safety zones (wall zone, corner zones), food zone and open zone. For the EPM task, the following predictors were used: occupation of the open arms, closed arms and center zone. If the independent variables (predictors) of the linear model fitted to the activity of a cell were significant (*P* < 0.05), the cell was classified as ‘modulated’ by the significant predictor, and ‘nonresponsive’ if not (*P* > 0.05). For significant predictors, positive regression coefficients defined ‘excited’ cells, and negative regression coefficients defined ‘inhibited’ cells, for example, ‘RW-excited’ or ‘RW-inhibited’ cells. In addition, for the ABA task in the relatively small enclosure, we set the required effect size to be above (excited cells) or below (inhibited cells) 0.1 s.d. from baseline. The Benjamini–Hochberg procedure was used to correct for multiple comparisons.

#### Estimation of overlap between responses

To evaluate the overlap of stimulus-evoked responses between anxiety tests (EPM, NSFT), we estimated the kernel densities of responses across the population (‘stats’ package, R) and assessed the proportion of cells with overlapping responses between both tests (‘overlap’ package, R).

#### Identification of PFC-modulated cells

For combined Ca^2+^ imaging of LepR^LH^ cells and optogenetic activation of cortical inputs into LH, we *z* scored the activity of individual cells across the whole recording session and averaged LepR^LH^ activity during ON and OFF phases of stimulation. To identify LepR^LH^ cells that were excited or inhibited by stimulation of PFC inputs, we applied a linear model (as mentioned above) to fit the Ca^2+^ signal of individual cells using the stimulation phase as a predictor. If the stimulation phase fitted to the activity of a cell was significant (*P* < 0.05), the cell was classified as ‘modulated’ by the stimulus phase, with positive or negative predictors defining ‘excited’ or ‘inhibited’ cells, respectively.

#### Prediction of ABA stage

To test whether the activity of cells in the ABA predicted ABA stage (habituation versus restriction), we averaged and *z* scored the Ca^2+^ signal of individual cells for each zone (food, RW and neutral zone) from eight animals. We built a support vector machine with radial basis function kernel (tunelength = 8, *σ* = 0.87, *C* = 1) and trained it on 70% of the original dataset to classify ABA stage with tenfold cross-validation (‘caret’ package, R). Accuracy of the model was estimated by applying the model to the test dataset and counting correct predictions. No information rate reflects the number of correct predictions when selecting the most common class, that is, the stage with more cells (restriction).

#### Prediction of anxiety category

To test whether the activity of cells in the restriction stage of the ABA predicted anxiety category (high versus low anxiety), we averaged and *z* scored the Ca^2+^ signal of individual cells from six animals for all RW visits from 3-s pre-entry to 6-s postentry. All animals were categorized as high-anxiety or low-anxiety animals based on the latency to eat in NSFT. We built a support vector machine classifier with radial basis function kernel (tunelength = 8, *σ* = 0.43, *C* = 32) and trained it on 80% of the original dataset to classify anxiety category with tenfold cross-validation (as mentioned above).

#### PCA

We first longitudinally registered individual LepR^LH^ neurons recorded during both phases of the ABA model and the EPM paradigm. To generate the set of variables for the covariance matrix, we averaged stimulus-evoked responses per cell (that is, open arm and closed arm in the EPM, RW and food in the ABA) per paradigm and stage of the ABA and normalized values across the population. PCA was performed using the ‘princomp’ function (‘stats’ package, R) to compute eigenvalues and eigenvectors from the covariance matrix, as determined by the cor function (‘stats’ package, R). We extracted the matrix of variable loadings and scores, and visualized the contribution of each variable using the ‘fviz_pca_var’ function (‘factoextra’ package, R) to generate biplots. Arrows of the biplot represent original variables projected onto principal component space.

### scRNA-seq data analysis of LH neurons

We conducted a transcriptomic analysis of neurons in the LH of females (LH neurons) using scRNA-seq data from dataset GSE188646 (ref. ^15^). Preprocessing and primary analysis steps were conducted using Seurat (v4.1.1). Doublet analysis was carried out using scDblFinder (v1.10.0).

#### Cell-type selection and standard workflow execution

We retrieved the Seurat object and isolated cells annotated as ‘neuron’ in the dataset’s metadata. These cells were processed using Seurat’s default normalization, scaling and PCA methods. Dimensionality reduction was performed using the RunUMAP function, which uses the UMAP algorithm. We selected the top 30 principal components—those explaining the highest variance—based on visual inspection of an elbow plot displaying changes in s.d. across 100 principal components. The remaining 70 components were disregarded due to their minimal contribution to dataset variability. To delineate distinct cellular populations, the shared nearest neighbor algorithm was used through Seurat’s FindNeighbors function within the defined 30D space. Clustering was performed using Seurat’s FindClusters function at a resolution of 0.5. The doublet analysis workflow of scDblFinder with default parameters was applied, successfully identifying and removing 1,310 doublet cells.

#### Marker gene analysis and Lepr^+^ cluster selection

High-confidence *Lepr*^+^ and *Lepr*^*−*^ clusters were identified through a multilevel approach. Initially, markers for each neuronal cluster were identified using logistic regression and the Wilcoxon rank-sum test through Seurat’s FindAllMarkers function. Clusters exhibiting differential expression (avg_log_2_(FC) > 0.2 and *P* < 0.05) by both methods were designated as *Lepr*^+^ cluster candidates.

#### Module score computation and high-confidence cluster validation

To confirm the identities of *Lepr*^+^ clusters and identify high-confidence *Lepr*^*−*^ clusters, neuronal cells, we performed a differential expression analysis between all *Lepr*^+^ cells and all *Lepr*^*−*^ cells using Seurat’s FindMarkers function. This enables the detection of enriched gene modules in each cell type. Gene module scores were then calculated for identified genes demonstrating a log_2_ fold change greater than 0.58 for *Lepr*^+^ cells and less than −0.58 for *Lepr*^*−*^ cells using Seurat’s AddModuleScore function. This approach quantifies gene expression coherence of a gene module within each cell against the background, providing a reliable metric for cellular identity. Clusters characterized by very low *Lepr*^−^ scores and high *Lepr* module scores were conclusively identified as *Lepr*^+^. For each cluster, *t* tests were additionally used to further affirm the significance of the differences between *Lepr*^+^ scores and *LepR*^−^ scores. All four clusters identified in the initial marker analysis (clusters 11, 20, 21 and 24) were confirmed as *Lepr*^+^ and showed highly significant differences between the gene modules, as envisioned. In addition, the top four clusters (2, 14, 20 and 28) identified as negative through *t* tests were selected for further analysis. We then reapplied the Seurat workflow on this refined and reduced dataset, selecting 40 principal components and a resolution of 0.02 and compared *Lepr*^+^ and *Lepr*^−^ cells globally for a second time. This refined approach yielded six clusters in total, along with many more markers, indicating an enhanced separation of the cells compared to the original dataset. These markers were then used to compute *Lepr*^+^ module scores for each cell, referred to as the confidence score. Violin plots were generated using Seurat’s ‘VlnPlot’ function with default quantitative parameters to visualize differential gene expression.

### Quantification and statistical analysis

Neuronal activity and fiber photometry data were analyzed in R (v4.3.3), while behavioral experiments were analyzed with GraphPad Prism (v9.3.1; GraphPad Software). Behavioral tracking data were exported from ANY-maze (v7.14; Stoelting) as csv files and organized in Excel (Excel 2016; Microsoft). Statistical tests were conducted using GraphPad Prism (v9.3.1; GraphPad Software) or R (v4.3.1), respectively, according to the experimental design and data structure. We used the Shapiro–Wilk test to test whether the data were normally distributed. For parametric data, we used Student’s *t* test or paired samples *t* test for paired designs. For comparisons across more than two groups, we applied ANOVA. In case of missing values, a restricted maximum likelihood mixed model as implemented in GraphPad Prism 8.0 was fitted. For nonparametric data, we used Mann–Whitney *U* test or Wilcoxon signed-rank test for paired designs. Multiple comparisons were adjusted for with Bonferroni correction. We exclusively used two-tailed parametric and nonparametric tests, where applicable. To assess the relationship of continuous variables, we used linear regression to fit the data and calculated Pearson’s R to evaluate the strength of the relationship. To compare proportions of neurons across conditions, we used *X*^2^ test. To compare proportion of neurons within conditions, we used the two-sample *Z* test. To test whether prediction accuracy achieved with a model was different from the no information rate, we performed an exact binomial test. For a statistical comparison between correlations, we used Fisher’s z transformation (‘cocor’ package, R). Plots show mean ± s.e.m., unless otherwise stated. To visualize the distribution of Ca^2+^ transients over time, we applied local polynomial regression (using the ‘loess’ function in the ‘stats’ package of R) or generalized additive models for large datasets (using the ‘gam’ function in the ‘mgcv’ package of R). To identify differentially expressed genes, we performed a Wilcoxon rank-sum test with Bonferroni multiple test correction using Seurat’s default ‘FindMarkers’ function. We set the significance threshold at 0.05. No statistical methods were used to predetermine sample sizes; sample sizes were chosen based on previous similar studies^39,44^. Experiment conditions were not blinded. Behavioral tracking was automated. All preprocessing steps were performed according to the predefined routine in batch mode, with the animal condition being blinded. Data analysis was subsequently performed blindly, using automatic data selection from a database. No animals or data points have been excluded from the analyses.

### Reporting summary

Further information on research design is available in the Nature Portfolio Reporting Summary linked to this article.

## Online content

Any methods, additional references, Nature Portfolio reporting summaries, source data, extended data, supplementary information, acknowledgements, peer review information; details of author contributions and competing interests; and statements of data and code availability are available at 10.1038/s41593-025-02078-y.

## Supplementary information

Reporting Summary

## Source data

Source Data Figs. 1–6 and Extended Data Figs. 1–6 Statistical source data.

## Data Availability

Source data underlying Figs. 1–6 and Extended Data Figs. 1–6 are provided with the paper. Source data are provided with this paper.

## Code availability

All code used in the current study is available from corresponding authors upon reasonable request.
