# Supplementary material for: A lateral hypothalamic neuronal population expressing leptin receptors counteracts anxiety to enable adaptive behavioral responses
Source: Nat Neurosci. 2025 Oct 20;28(11):2262–72. doi: 10.1038/s41593-025-02078-y (PMC12586148; doi:10.1038/s41593-025-02078-y)
Supplement: Supplementary file 1 — Reporting Summary [file 41593_2025_2078_MOESM1_ESM.pdf]

nature portfolio

Corresponding author(s): T. Korotkova, A. Petzold

Last updated by author(s): Aug 19, 2025

Reporting Summary

Nature Portfolio wishes to improve the reproducibility of the work that we publish. This form provides structure for consistency and transparency in reporting. For further information on Nature Portfolio policies, see our Editorial Policies and the Editorial Policy Checklist.

Statistics

For all statistical analyses, confirm that the following items are present in the figure legend, table legend, main text, or Methods section.

n/a	Confirmed	
☐	☒	The exact sample size (*n*) for each experimental group/condition, given as a discrete number and unit of measurement
☐	☒	A statement on whether measurements were taken from distinct samples or whether the same sample was measured repeatedly
☐	☒	The statistical test(s) used AND whether they are one- or two-sided<br>*Only common tests should be described solely by name; describe more complex techniques in the Methods section.*
☐	☒	A description of all covariates tested
☐	☒	A description of any assumptions or corrections, such as tests of normality and adjustment for multiple comparisons
☐	☒	A full description of the statistical parameters including central tendency (e.g. means) or other basic estimates (e.g. regression coefficient) AND variation (e.g. standard deviation) or associated estimates of uncertainty (e.g. confidence intervals)
☐	☒	For null hypothesis testing, the test statistic (e.g. *F*, *t*, *r*) with confidence intervals, effect sizes, degrees of freedom and *P* value noted<br>*Give P values as exact values whenever suitable.*
☒	☐	For Bayesian analysis, information on the choice of priors and Markov chain Monte Carlo settings
☒	☐	For hierarchical and complex designs, identification of the appropriate level for tests and full reporting of outcomes
☐	☒	Estimates of effect sizes (e.g. Cohen's *d*, Pearson's *r*), indicating how they were calculated

Our web collection on statistics for biologists contains articles on many of the points above.

Software and code

Policy information about availability of computer code

Data collection	Inscopix Data Acquisition software for single cell calcium imaging data;<br>Doric Neuroscience Studio for fiber photometry imaging data;<br>ANY-maze or Pylon for behavioral video acquisition;<br>Leica Application Suite X (LAS X) software for confocal imaging, EVOS for wide-field imaging.
Data analysis	R (v4.3) or GraphPad Prism 9 (GraphPad Software) for plotting figures and statistical analysis;<br>Fiji - ImageJ for analysing confocal and widefield images;<br>ANY-maze for automated behavioral tracking, DeepLabCut toolbox (version 2.2.0.2) for tracking in EPM test;<br>BORIS for manual scoring of behavior;<br>Adobe Illustrator 2020 for assembling figures.<br>CellPose (v3.0.10) for analysing confocal images

For manuscripts utilizing custom algorithms or software that are central to the research but not yet described in published literature, software must be made available to editors and reviewers. We strongly encourage code deposition in a community repository (e.g. GitHub). See the Nature Portfolio guidelines for submitting code & software for further information.

Data

Policy information about availability of data

All manuscripts must include a data availability statement. This statement should provide the following information, where applicable:

- Accession codes, unique identifiers, or web links for publicly available datasets
- A description of any restrictions on data availability
- For clinical datasets or third party data, please ensure that the statement adheres to our policy

Datasets generated during the current study are available from corresponding authors upon reasonable request.

Human research participants

Policy information about studies involving human research participants and Sex and Gender in Research.

Reporting on sex and gender	n/a
Population characteristics	n/a
Recruitment	n/a
Ethics oversight	n/a

Note that full information on the approval of the study protocol must also be provided in the manuscript.

Field-specific reporting

Please select the one below that is the best fit for your research. If you are not sure, read the appropriate sections before making your selection.

☒ Life sciences ☐ Behavioural & social sciences ☐ Ecological, evolutionary & environmental sciences

For a reference copy of the document with all sections, see nature.com/documents/nr-reporting-summary-flat.pdf

Life sciences study design

All studies must disclose on these points even when the disclosure is negative.

Sample size	Sample size was chosen according to similar previously published studies reflecting standards in the field (e.g. Lee et al., 2023, Ref. 39; Giardino et al., 2018, Ref. 44).
Data exclusions	No data were excluded from analysis.
Replication	All attempts at replication were successful.
Randomization	Animals were randomly assigned to control and experimental groups.
Blinding	Manual scoring of behavior was performed blinded, and behavioral tracking was automated. All preprocessing steps were run according to the pre-defined routine in batch mode, blind to animal condition. Data analysis was subsequently performed blindly using automatic selection of data from a database.

Reporting for specific materials, systems and methods

We require information from authors about some types of materials, experimental systems and methods used in many studies. Here, indicate whether each material, system or method listed is relevant to your study. If you are not sure if a list item applies to your research, read the appropriate section before selecting a response.

Materials & experimental systems

n/a	Involved in the study
☐	☒ Antibodies
☒	☐ Eukaryotic cell lines
☒	☐ Palaeontology and archaeology
☐	☒ Animals and other organisms
☒	☐ Clinical data
☒	☐ Dual use research of concern

Methods

n/a	Involved in the study
☒	☐ ChIP-seq
☒	☐ Flow cytometry
☒	☐ MRI-based neuroimaging

Antibodies

Antibodies used	Primary antibodies against ebf1 (Sigma-Aldrich), AB10523, polyclonal, 1:200, were used. Secondary antibodies anti-rabbit IgG H&L (Alexa Fluor® 488) (Abcam) AB150077, 1:500 were used
Validation	Anti-ebf1 has been validated to show species reactivity for human and mouse, can be used for Western Blotting, IHC(P), Immunofluorescence, Dot Blot, and is recommend to be used in a dilution of 1:200-1:1000 for immunofluorescence (see statement on manufacturer's website: https://www.merckmillipore.com/DE/de/product/Anti-EBF-1-Antibody,MM_NF-AB10523, as well as Ref. Timblin, Schlissel, 2013, https://doi.org/10.4049/jimmunol.1301675). In the manuscript histology pictures are shown in Fig. 4d and Ext. Data Fig. 5d, and a description of method is given in the methods section "Immunohistochemistry".

Animals and other research organisms

Policy information about studies involving animals; ARRIVE guidelines recommended for reporting animal research, and Sex and Gender in Research

Laboratory animals	LepR-Cre knock-in mice, LepR-flox mice, Nts-Cre knock-in mice (The Jackson Laboratory, Bar Harbour, USA) and C57BL/6 mice, 12 - 25 weeks old, were used.
Wild animals	No wild animals were used this study.
Reporting on sex	Male and female mice were used in this study.
Field-collected samples	No field collected samples were used in this study.
Ethics oversight	All animal procedures were performed in accordance with national and international guidelines and were approved by the local health authority (LANUV, Das Landesamt für Natur, Umwelt und Verbraucherschutz).

Note that full information on the approval of the study protocol must also be provided in the manuscript.
